# Emergence and Prevalence of Human Vector-Borne Diseases in Sink Vector Populations

**DOI:** 10.1371/journal.pone.0036858

**Published:** 2012-05-18

**Authors:** Guilhem Rascalou, Dominique Pontier, Frédéric Menu, Sébastien Gourbière

**Affiliations:** 1 UMR 5244 CNRS-UPVD Ecologie et Evolution des Interactions, Université de Perpignan Via Domitia, Perpignan, France; 2 UMR 5558 CNRS-UCBL Laboratoire de Biométrie et Biologie Evolutive, Université de Lyon, Université Lyon 1, Villeurbanne, France; 3 School of Life Sciences, University of Sussex, Brighton, United Kingdom; University of Oxford, Viet Nam

## Abstract

Vector-borne diseases represent a major public health concern in most tropical and subtropical areas, and an emerging threat for more developed countries. Our understanding of the ecology, evolution and control of these diseases relies predominantly on theory and data on pathogen transmission in large self-sustaining ‘source’ populations of vectors representative of highly endemic areas. However, there are numerous places where environmental conditions are less favourable to vector populations, but where immigration allows them to persist. We built an epidemiological model to investigate the dynamics of six major human vector borne-diseases in such non self-sustaining ‘sink’ vector populations. The model was parameterized through a review of the literature, and we performed extensive sensitivity analysis to look at the emergence and prevalence of the pathogen that could be encountered in these populations. Despite the low vector abundance in typical sink populations, all six human diseases were able to spread in 15–55% of cases after accidental introduction. The rate of spread was much more strongly influenced by vector longevity, immigration and feeding rates, than by transmission and virulence of the pathogen. Prevalence in humans remained lower than 5% for dengue, leishmaniasis and Japanese encephalitis, but substantially higher for diseases with longer duration of infection; malaria and the American and African trypanosomiasis. Vector-related parameters were again the key factors, although their influence was lower than on pathogen emergence. Our results emphasize the need for ecology and evolution to be thought in the context of metapopulations made of a mosaic of sink and source habitats, and to design vector control program not only targeting areas of high vector density, but working at a larger spatial scale.

## Introduction

Vector-borne diseases represent one of the biggest challenges to the current and future human wellbeing [1], [2]. Various insects are responsible for the transmission of the well-known malaria, West-Nile virus, yellow fever, Japanese encephalitis, as well as a cluster of so-called ‘neglected tropical diseases’ such as dengue, leishmaniasis, human American and African trypanosomiasis [3]. All these diseases have severe impacts on many tropical and subtropical countries, where they are responsible for around 10% of human deaths [4]–[7], and contribute substantially to impoverishment by imposing annually a burden of more than 50 million of disability-adjusted life years (DALYs) [4]–[9]. Vector-borne diseases are also becoming a serious health-concern for more developed countries [10]–[13], because of the expansion of vectors geographic distribution in response to climatic changes [14]–[19], or the accidental introductions of vectors or pathogens through increasing international migration and commercial exchanges [20]–[23].

A large body of empirical and theoretical studies on human vector-borne diseases has contributed to our understanding of the importance of vectors ecology and evolution in disease transmission (e.g., [24]), pathogen evolution (e.g., [25]) and the design of efficient control strategies [26]. These studies typically focus on highly endemic areas, where pathogens are transmitted by large self-sustaining ‘source’ populations [27], [28] of key vectors of human diseases; mosquitoes (*Anopheles*, [29], *Aedes*, [30], or *Culex*, [31]), flies (*Glossina*, [32], and phlebotomines, [33]), or triatomines (*Triatoma infestans*, [34], [35], and *Rhodnius prolixus*, [36]).

However, vector populations can also be ‘sink’ populations wherever the environment does not provide suitable conditions for reproduction or survival of individual vectors, so that such ‘sink’ populations cannot sustain themselves and have to be sustained by immigration [27], [28]. Sink populations have been described for the vectors of human African trypanosomiasis [37], Chagas disease [38], and malaria [39]. Although much less attention has been paid to such populations, they are likely to play a significant role in the transmission of vector borne diseases. In highly endemic areas, vector control is a key strategy to lower the impact of those diseases on humans [3], [40] through chemical [41]–[43] or biological control [44]–[46]. However such campaigns are unavoidably restricted in their local efficacy and/or spatial coverage [47], [48], so that partially controlled populations effectively constitute ‘anthropic’ sinks sustained by immigration from wild or non-targeted areas [49]–[54]. Vector populations can also be ‘natural’ sinks either in the core of their niche, when the habitat is heterogeneous, or at the border of the niche [37], [55]–[57]. Such populations will be the typical pathogen environment where vectors spatial distributions are expanding following environmental changes [17]–[19]. A better knowledge of pathogen transmission in sink vector populations is thus critically needed to address two main challenges to human health associated with vector-borne pathogens: the persistence of transmission in highly endemic areas despite ongoing vector control programs [35], [58], and the prediction of the risk of disease emergence in areas where vectors are expanding because of environmental changes [14], [59], [60].

The spread of vector-borne pathogens is commonly thought to critically rely on vector demography and feeding rates (e.g., [61]). In sink populations, vector immigration and local (negative) growth rate will undoubtedly be two key demographic processes, since species abundance has repeatedly been demonstrated to depend on the balance between them [27], [28]. In such populations one can also anticipate that, given the low vector abundance, the number of contacts each individual is able to make with hosts will have a critical impact on transmission. A quantitative assessment of such qualitative predictions requires to tightly link transmission and the two main determinants of vector feeding; the minimal amount of time elapsing between two blood-meals (e.g., [61]), and the host availability and accessibility (e.g., [62]). Clearly, the fate of vector-borne pathogens in sink vector populations will also depend on the ease of the transmission when contacts are established, and on the within-host dynamics of the pathogens. Critically, those last two determinants of disease dynamics show significant variations among human vector-borne diseases (e.g., [24]). Unfortunately, the typically low vector abundances encountered in sinks make it difficult to set up field experiments to look at these different components of vector transmission in such populations [63], [64].

In the present work, we aim to produce theoretical insights into key human pathogens’ transmission in sink vector populations. Our general objective is to identify the key processes determining the emergence and subsequent prevalence of pathogens in such vector populations to help specifying priority targets for future field studies. We adopted an approach inspired from [65] that consists of developing a unique ‘core model’ including the main processes described above and involved in the transmission of major human vector-borne diseases, not accounting for the more disease-specific processes, such as seasonal forcing, host or pathogen diversity and heterogeneity, which would divert from drawing general conclusions and limit cross comparisons between diseases [65]. We developed a SIRS model (‘Susceptible-Infected-Recovered-Susceptible’, e.g., [66] p. 247), which provides a simple description of the key processes of vector demography and feeding that we identified above, as well as of pathogen transmission and virulence. This ‘core model’ includes human and alternative hosts, thereafter generically referred to as ‘non-human hosts’, as these non-human hosts can have profound effects on disease dynamics when the pathogen is not specific to humans (e.g., [67]). Since a systematic analysis of the model would be rather cumbersome, and irrelevant in most of the highly dimensional parameter space, we focused on six human diseases that, not only represent major public health concerns, but also show contrasted patterns regarding the existence or absence of non-human hosts, their vector’s life-history and feeding rate, and the transmission and within-host dynamics of their causal agents.

Importantly, there are two different ways for vector immigration to influence the pathogen transmission [68]. When immigrating vectors carry on the pathogens, they can have a direct effect not only on vector abundance, but also on pathogen transmission. Such a situation has been documented when tsetse flies [69], sandflies [55] or triatomines [70]–[72] infest human habitat bringing in the pathogens. Immigration of non-infectious vectors can also contribute to build-up a susceptible vector population, where pathogens can subsequently be introduced by the arrival of, e.g. mammals, hosts from endemic areas. It has indeed been shown that both human [73], [74] and non-human hosts [23], [75] have been the cause of pathogens’ introduction or re-introduction. We thus investigated separately these two epidemiologically very different situations within our ‘core model’.

## Materials and Methods

### Human Vector-borne Diseases

We considered three diseases with only human hosts; malaria (MAL), dengue (DEN), and the Gambian form of human African trypanosomiasis (HAT), which all together affect over 250 millions people and kill around 900,000 humans every year [4], [5], [7]. We also included three diseases with non-human hosts; Japanese encephalitis (JE), American trypanosomiasis, often called Chagas disease (CD), and visceral leishmaniasis (VL). Those additional diseases are responsible for more than 50,000 human deaths a year, and incapacitate several hundred thousands people [4], [7]. Detailed descriptions of these diseases can be found in specialized books (see [76]–[81] for MAL, DEN, HAT, JE, CD and VL, respectively). Below we provide a brief summary of the main differences in the characteristics of their vectors, non-human hosts and pathogens, which were quantified by reviewing the literature. S1 provides a detailed description of the origin of the data and procedures used to obtain estimates of all parameters appearing in Table 1.

**Table 1:** Parameters definition and range of values.

Parameter definition	Symbol	Dimension	MAL	DEN	HAT	VL	JE	CD
**Vector demography and feeding**								
Vector life expectancy (1)	1/Δ*_V_*	days	1–15	1–15	1–45	1–15	1–15	1–210
Number of immigrants	*i_V_*	ind.day^−1^	]0, 67]	]0, 67]	]0, 22]	]0, 67]	]0, 67]	]0, 5]
Fraction of infectious immigrants	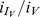	–	]0, 0.02]	]0, 0.02]	]0, 0.02]	]0, 0.02]	]0, 0.02]	]0, 0.35]
Minimal delay between blood-meals	*T_d_*	days	]2, 6]	]2, 6]	]2, 6]	]2, 6]	]2, 6]	]7, 28]
Finding rate	*a*	day^−1^	]0, 1]	]0, 1]	]0, 1]	]0, 1]	]0, 1]	]0, 1]
**Host demography**								
Human abundance	*N_H_*	ind.	1000	1000	1000	1000	1000	1000
Non-human hosts abundance	*N_h_*	ind.	–	–	–	1000/6	1000/6	1000/6
Human natural life expectancy (1)	1/*d_H_*	years	60	60	60	60	60	60
Non-human natural life expectancy (1)	1/*d_h_*	years	–	–	–	3	1	3
**Pathogen transmission**								
From vector to human hosts	*p_HI_*	–	0.01–0.13	0.50–1	0.50–0.70	0.20–0.40	0.01–0.04	0.6e^−3^–3.8e^−3^
From vector to non-human hosts	*p_hI_*	–	–	–	–	0.20–0.40	0.27–0.45	0.6e^−3^–3.8e^−3^
From infectious human to vector	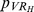	–	0.24–0.64	0.15–0.73	1.7e^−3^–25e^−3^	0.21–0.29	0.14–0.38	0.90–0.99
From ‘recovered’ human to vector	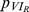	–	0.024–0.064	0	0	0	0	4.2e^−3^–6.2e^−3^
From infectious non-human hosts to vector	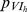	–	–	–	–	0.05–0.28	0.55–1	0.90–0.99
From ‘recovered’ non-human hosts to vector	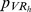	–	–	–	–	0	0	0.05–0.31
**Pathogen within-host dynamics**								
Infectious human death rate (2)	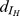	day^−1^	0.4e^−4^–4.9e^−4^	0.4e^−4^–67.8e^−4^	0.4e^−4^	2.7e^−4^–4.3e^−2^	37.1e^−4^–0.26	0.4e^−4^–11.9e^−4^
‘Recovered’ human death rate	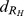	day^−1^	0.4e^−4^	0.4e^−4^	73.3e^−3^–3.8e^−2^	0.4e^−4^	0.4e^−4^	0.4e^−4^–64.0e^−4^
Infectious human rate of recovery	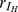	day^−1^	15.9e^−4^–1.7e^−2^	6.6e^−2^–0.33	12.8e^−4^–83.3e^−4^	55.6e^−4^–1.1e^−2^	7.1e^−2^–0.50	1.7e^−2^–2.2e^−2^
Human rate of loss of immunity	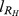	day^−1^	0–1.1e^−2^	0.4e^−4^	13.7e^−4^–83.3e^−4^	0	0	0
Infectious non-human hosts death rate (2)	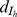	day^−1^	–	–	–	51.2e^−4^–4.61	27.4e^−4^–4.61	9.1e^−4^–20.5e^−4^
‘Recovered’ non-human hosts death rate	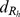	day^−1^	–	–	–	9.1e^−4^	27.4e^−4^	9.1e^−4^–12.8e^−4^
Infectious non-human hosts rate of recovery	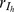	day^−1^	–	–	–	9.1e^−4^–1	0.14–1	1.3e^−2^–2.2e^−2^
Non-human hosts rate of loss of immunity	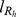	day^−1^	–	–	–	1	0	0

(1) Vector, human and non-human hosts natural death rates were estimated as 1/individual longevity. The range of variation of longevity (i.e. 1/death rate parameter defined in the model), as those are the raw data found in the literature (see sections ‘*Vector local growth rate*’ and ‘*Human and non-human hosts natural death rates*’ in Text S1).

(2) Death rates were calculated as the sum of the natural death rate of human 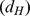 or non-human 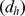 hosts and additional mortality imposed by the pathogen to infectious and ‘recovered’ individuals (as calculated in section ‘*Human and non-human hosts mortality induced by the pathogen*’ in Text S1).

#### Diseases with only human hosts

MAL and DEN are two diseases transmitted by mosquitoes, while the vectors of HAT are tsetse flies. Mosquitoes and tsetse flies have similar average frequency of feeding (around 3 days), but tsetse flies tend to have longer adult life-expectancy than mosquitoes (around 2 vs. 6 weeks) so that individuals can bite around 15 times vs. 5 for mosquitoes, during the hematophagous stage of their life-cycle. On the contrary, the transmission potential is lower for tsetse flies (around 0.008) than for mosquitoes transmitting MAL (0.003–0.03 depending on the status of human host, see below) and DEN (around 0.3). This transmission potential was defined as the product of the probabilities of transmission from vector to host and from host to vector, and was calculated from the median of the range of parameter values that appear in Table 1. These three diseases also differ in the way pathogens afflict their hosts. For MAL and DEN, individuals first go through an infectious state, which can last from a few days for DEN and up to several months for MAL. Individuals infected with DEN can then recover and acquire a life-long immunity, while hosts infected with MAL enter a state of reduced infectivity [82], [83] and eventually return to a susceptible state after a few months or years. The course of HAT is more singular. Infected hosts first enter an asymptomatic state, usually called ‘phase 1’, followed by a symptomatic state, called ‘phase 2’, both of which lasting several months. Individuals in phase 1 are infectious, while those in phase 2 are usually considered as non-infectious, all the more as they may be under treatment. Further, phase 2 is eventually fatal for humans not pursuing treatment, and those surviving this phase do not acquire immunity but return to the susceptible pool. Finally, disease-induced mortality is higher for HAT than for MAL and DEN.

#### Diseases with Non-human Hosts

JE, CD, and VL show significant differences in their vector and pathogen’s within-host dynamics. Sandflies have similar feeding frequency (around 3 days) and life-expectancy (around 2 weeks) to mosquitoes, but triatomines are very unusual vectors. Although they feed less frequently (around 1–4 weeks), adults live for several months so that they can bite 10–30 times. The transmission potential between vectors and human hosts is larger for VL (around 0.08) than for JE (around 0.007) and for CD (around 0.002 and 1.10^−5^ for human hosts with acute and chronic infection, respectively). The transmission potential between vector and non-human hosts shows a similar trend, with larger probabilities for JE (around 0.28) than for VL (around 0.05) and for CD (around 0.002 and 4.10^−4^ for non-human hosts with acute and chronic infection, respectively). The course of the disease in hosts also differs between the three diseases. Human hosts affected by VL and JE go through an acute and infectious state that last a few days for JE, or up to several months for VL. Once they have recovered, individuals are immune for the rest of their life. Disease-induced death rate during the infectious state can be very high for both diseases, and humans suffering from VL will eventually die if not treated. JE, CD and VL’s pathogens are known to circulate in various non-human hosts, although an understanding of the pathogens’ development in those hosts remains limited. Here, we focused on emblematic domestic hosts, dogs for VL and CD and swine for JE, as they are claimed to be key actors regarding transmission, and they are central to control strategies set up to limit the impact of these diseases. The course of VL in dogs or JE in swine is roughly similar, except that infected dogs do not usually recover and remain infectious until death, which can be natural, induced by the disease, or due to euthanasia. The progress of CD in (human or dog) hosts is different from the course of VL and JE (in humans, dogs and swine). An acute phase, lasting several weeks, is followed by a chronic and life-long phase and hosts are infectious in both phases, although parasitemia is significantly lower in the chronic stage of the disease [80].

### Modelling

#### The SIRS model

We developed a SIRS model ([66] p. 247) to study the vector transmission of a pathogen between human and non-human hosts. The complete model was used to investigate diseases with non-human hosts (JE, CD, and VL), and the number of such hosts was set to 0 when considering diseases with only human hosts (MAL, DEN and HAT). In our complete model, human and non-human hosts can be susceptible (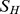,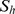), infectious (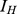,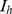) or belong to a last category (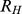,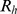), whose exact meaning varies with the modelled disease. Human hosts falling in this last category are thought to be *recovered* and immune when considering DEN [84]. When modelling MAL and HAT individuals with status 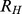 still carry the pathogen, but are *removed* from the infectious category as they become much less able [82], [83] or unable to transmit [85]. For JE and VL, human (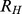) and non-human 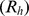 individuals are thought to have *recovered* and be immune to new infection [31], [86]. Finally, when considering CD, infectious human and non-human hosts are individuals in the acute phase of the disease, while 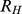 and 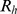 individuals have entered the chronic phase, where there are fewer circulating pathogens but hosts remain able to transmit [67]. Effectively, for all diseases, individuals thereafter commonly referred to as ‘recovered’, are thus either not or much less able to transmit the pathogen than when they are infectious.

Human host population size is assumed to be constant, and equal to 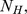 so that only the numbers of infectious and ‘recovered’ are modelled explicitly. Infectious humans die at rate 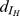 (which includes natural death, 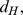 and disease-induced mortality of infectious human hosts, 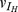), become ‘recovered’ at rate 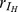, and are gained through contacts of susceptible individuals with infectious vectors (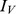) at rate 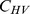 (see section ‘*Modelling transmission with respect to vector feeding’* for a formal expression). This leads to a first ordinary differential equation:
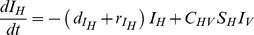
(1)


‘Recovered’ humans die at rate 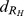 (which includes natural death, 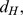 and disease-induced mortality of ‘recovered’ humans, 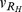), and can re-join the pool of susceptible by losing their immunity (for MAL and DEN) or after treatment (for HAT) at rate 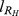. This leads to a second ordinary differential equation:
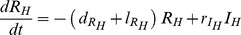
(2)


The non-human host population is also assumed to be constant (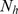), and is modelled exactly in the same way as the human host population, although demographic and transmission parameters are allowed to take on specific values. This leads to define two additional ordinary differential equations:
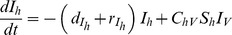
(3)

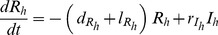
(4)where 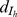 (which includes non-human hosts natural death, 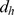, and disease-induced mortality of infectious non-human hosts, 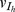), 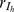, 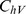, 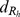 (which includes natural death, 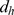, and disease-induced mortality of ‘recovered’ non-human hosts, 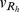), and 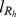 are defined as for the human host population.

By contrast to human and non-human hosts, both the number of susceptible and infectious vectors are modelled explicitly. Since we are interested in sink vector populations, the local growth rate of vectors is assumed to be negative (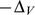). Such a local growth rate actually represents the net balance between vector’s births, deaths and emigration, and 1/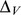 corresponds to the average time spent in the sink, or vector ‘longevity’ in the sink. Vector population is sustained by immigration of individuals (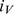), some being susceptible (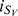), while others are infectious (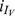). Neglecting vertical transmission, susceptible vectors become infectious only by contact with infectious and recovered human and non-human hosts at rate 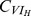, 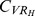 and 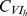, 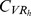, respectively (see section ‘*Modelling transmission with respect to vector feeding'*). The two ordinary differential equations describing the temporal variations of the vector population then read:
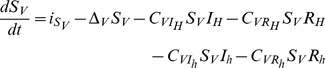
(5)

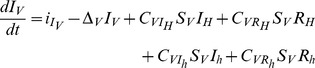
(6)


Altogether equations 1–6, where 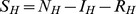 and 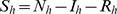, define our SIRS model.

#### Modelling transmission with respect to vector feeding

Key ingredients of any infectious disease model are the rates of transmission of the pathogen (noted 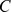 in our model). For vector-borne diseases, they usually are taken to be frequency-dependent, assuming that each vector bites at a constant rate [25], [24]. In this contribution, we aim to look at the importance of the vector feeding in determining this biting rate. We took advantage of an original function of transmission [87], which links explicitly the biting rate of the vector to two key ingredients of vector feeding through a couple of parameters. First, the proportion of the host population that has been found by a vector within a given time period, thereafter referred to as ‘finding rate’ of the vector (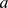), which accounts for various features of vector feeding behavior and host accessibility and availability. Second, the minimal amount of time between two consecutive blood-meals (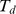). Using this function one can write the rate at which vectors become infected by contact with infectious humans:
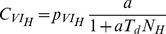
(7)where 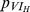 stands for the probability of transmission (per contact) from an infectious human host to a vector. Interestingly, when considering a long delay between blood-meals (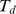) or a high finding rate (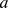), the Antonovics et al.’s function [87] tends towards a frequency-dependent function of transmission, while in case of a short delay (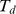) or a low finding rate (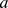), it becomes density-dependent. All the other rates of contact (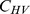,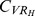, 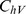, 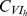, 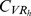) can be expressed exactly in the same way, but changing the probability of transmission (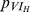 above) and the number of hosts (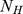 above), with respect to the type of human or non-human hosts being considered. This function of transmission does not account for any host preference. Such preferences have been documented for most vectors of human pathogens, although the pattern of vector feeding plasticity are still hard to measure and there is no general understanding of their ecological and evolutionary determinants [88]. Although host preference can have effects on transmission [89] and control [90] of multi-host pathogens, looking at these effects thus falls far behind the goal of this paper.

### Analysis

#### Dynamical properties of the model

We first investigated the dynamical properties of our model to determine conditions on vector demography and feeding rates as well as on pathogen transmission and virulence that allow for the spread and persistence of vector-borne diseases in sink vector populations. We identified the basic reproduction rate of the parasite, noted R_0_ (e.g., [91]), the steady states of the model, and evaluated their properties of local stability. The expression of the equilibrium levels of susceptible/infectious/recovered humans and alternative hosts were derived from basic methods to analyse second order polynomial equations, and Cardan’s method to solve cubic equations. The stability properties of these steady states were established using standard Routh-Hurwitz criterion [92].

#### Quantitative investigations of the spread and persistence of the pathogens

The expressions of the R_0_ or the level of prevalence of the pathogens in human populations derived from these analyses were then investigated quantitatively. Because studies on sink vector populations are rare (see introduction), we would not find estimates of all relevant parameters in a given field site (as it can be for well documented source populations, e.g., [30], [34]). This precluded us from performing standard sensitivity analysis in the vicinity of a trustable set of parameter values estimated on a specific population (e.g., [93]). Instead we used an approach developed by [94], which consists of generating random combinations of parameter values within the biologically plausible range of these parameters (rather than around specific estimates). In this way, we aimed at reproducing a representative set of biologically sensible conditions that could be encountered by different pathogens in various sink vector populations. We thus used the estimates of the parameters of the model that could be gained from our review of the literature (Text S1) to specify the biologically relevant subset of the parameter space to be looked at (Table 1).

We performed sensitivity analysis to identify which of the parameters most strongly influence the value of R_0_, and the prevalence in humans. For each modelled disease, we generated 10,000 sets of parameter values by randomly sampling each parameter within its identified range of plausible values according to a uniform distribution. The assumption that parameters are uniformly distributed has been used to model transmission in other contributions (e.g., [95], [96]). Potentially, considering alternative distributions could change the quantitative details of the results, though qualitative trends are likely to be robust as they reflect the basic features of the source-sink situation we modelled (see discussion). A uniform distribution is the simplest non-informative assumption that can be made according to the principle of ‘insufficient reason’ [96] in the absence of data supporting a specific pattern of variability, We then calculated the value of R_0_ and the prevalence in humans for each of the 10,000 sets of parameter values and used this to draw, for each disease, the distribution of the expected values of R_0_ and of human prevalence (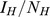 and 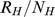) in sink vector populations. A great value of this approach is that the effect of a given parameter is quantified, while all other parameters are varied randomly within their range, rather than when they take on given estimated values.

The effect of a given parameter on R_0_ can then be quantified by *a posteriori* comparing the subsets of its values that were associated with R_0_>1 and with R_0_<1 in the 10,000 virtual populations that we generated by sampling the plausible range of parameter values [94]. If a parameter has a small effect on R_0_, one expects this parameter to take on similar values in populations where the pathogen spreads (R_0_>1) and in populations where it does not (R_0_<1). In the opposite situation, whereby a parameter has a strong effect on R_0_, small changes in its value will be sufficient to switch from a situation where the pathogen spreads to a situation where it gets extinct. Accordingly, the larger the effect of a parameter on R_0_, the lower the overlap between the distributions corresponding to the two subsets is expected to be. We thus calculated the proportion *p* of the two distributions that overlapped, and use 1−*p* as a measure of the effect of the parameter being considered.

The effect of a given parameter on the percentage of human individuals being infectious or recovered cannot be quantified as its effect on R_0_. As a matter of fact, in this case, one cannot define two subsets of values corresponding to two qualitatively different dynamical outcomes (such as, in the previous case, ‘spread’ corresponding to R_0_>1, vs ‘extinction’ corresponding to R_0_<1). Instead, we thus simply correlated the values of these percentages (calculated while sampling in all the range of parameter values) with the sampled values of the parameter being considered. We then used the coefficient of determination of the regression to the mean as a measure of the effect of the parameter on the percentage of infectious or recovered individuals, since it typically gives the proportion of the total variation of the dependent variable that is accounted for by the explanatory variable. The analytical expression of the equilibrium levels of susceptible/infectious/recovered human and non-human hosts were evaluated numerically for any given set of parameter values using Mathematica [97].

## Results

### Conditions for the Spread of Vector-borne Pathogens in Sink Vector Populations

The stability analysis of our model confirmed that the two epidemiological situations presented in introduction, whereby pathogens are introduced by immigrating vectors, or independently of vector immigration (i.e. via the accidental arrival of infected human or non-human hosts in the sink population), are very different from a dynamical system point of view. The dynamical behaviour of the model in these two situations is briefly summarized below.

#### Introduction of pathogens via immigrating vectors

Because a fraction of the immigrating vectors is infectious, both vector and pathogen will persist as soon as vector immigration into the sink population is present. As expected, there is then only one stable positive ‘endemic equilibrium (thereafter referred to as EE), where the pathogen infects human hosts and, when they are present, non-human hosts. A more formal way to express the conditions for pathogen persistence is to phrase it in term of R_0_, where R_0_ = 1+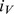, which indicates that the vector immigration threshold for the parasite to spread is 0. In this first situation, the spread of the pathogen thus does not depend on the various other parameters of the model.

#### Independent introduction of vectors and pathogens

In this second situation, there are two equilibria; a disease-free equilibrium (thereafter referred to as DFE) and the endemic equilibrium EE. As for most vector-borne disease models, we found a transcritical bifurcation, whereby 1) the DFE is unstable when the EE is stable (and vice versa), and 2) the DFE is unstable when the basic reproduction rate of the parasite R_0_ is larger than 1 (e.g., [91]). However, as the transmission process is modelled by using the Antonovics et al.’s function [87], an expression of R_0_ can be proposed that, according to the minimal amount of time between two blood-meals (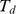) and the vector finding rate (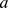), will be associated to either a density- or a frequency-dependent function of transmission [87]. The general expression of R_0_ in sink vector population then reads:
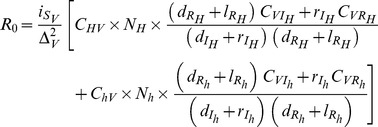
(9)


Straightforward calculations show that when considering long time between blood-meals or high finding rate (which makes the function of transmission frequency-dependent, as commonly modelled for vector-borne diseases), the R_0_ in sink vector population simplifies to:
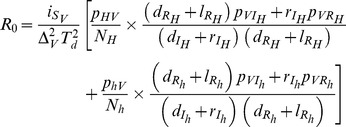
(10)


We note that substituting the immigration term (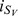) with a constant reproduction rate, this expression is similar to those derived for a source vector population (e.g., [25] page 16). From equation 9 (or 10) it is obvious to show that the persistence of a pathogen in a sink population sustained by the arrival of non-infectious vectors requires immigration to exceed a threshold, so that R_0_>1. This threshold depends on all other parameters describing vector demography and feeding rates, host demography, transmission and within-host dynamics (see Table 1) in various non-linear ways. The sensitivity analysis presented in the next paragraph will allow identifying which of these parameters play a key role in the spread of the 6 diseases considered in this study.

### Identification of the Key Processes Determining the Emergence and Prevalence of Vector-borne Pathogens in Sink Vector Populations

#### Rate of spread of pathogen in disease-free sink vector populations

The previous section has made explicit that, obviously, when some immigrating vectors are infectious, the pathogen will always persist in the sink population. Here, we will only look at the condition for the pathogen to spread when it is not introduced by immigrating vectors but by the incidental arrival of infected hosts (see equation 9). To determine the typical rates of spread in this second case, we generated the distribution of R_0_ for the six diseases considered by randomly sampling into each parameter range of plausible values (Table 1).

**Figure 1:**
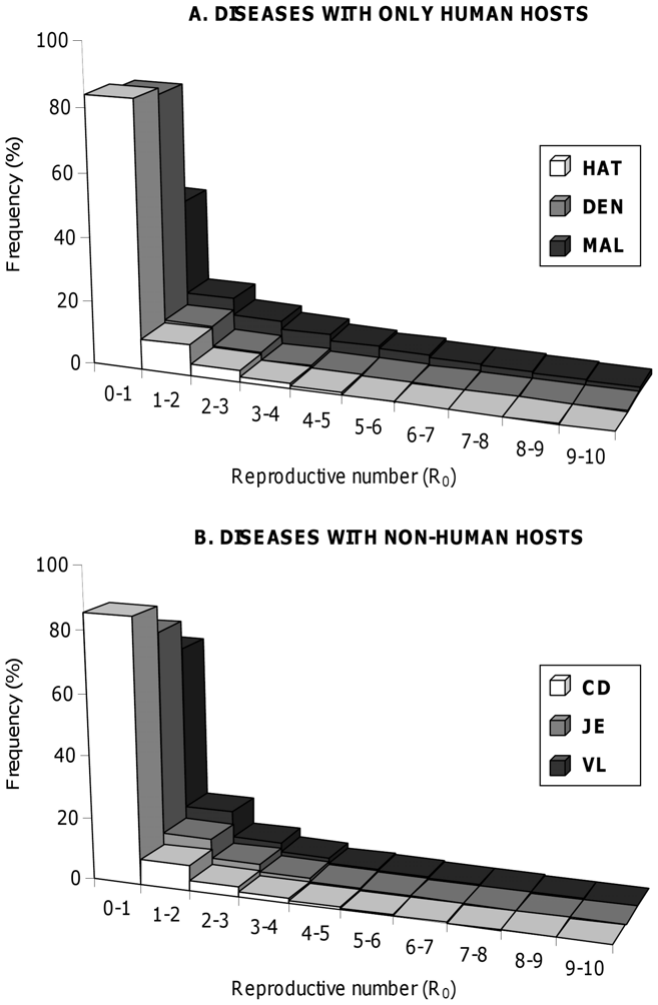
Distribution of the pathogen’s basic reproduction number (R_0_) for each of the six vector-borne diseases considered. (**A**) Diseases with only human hosts: human African trypanosomiasis (HAT), dengue (DEN) and malaria (MAL). (**B**) Diseases with non-human hosts: Chagas disease (CD), Japanese encephalitis (JE), and visceral leishmaniasis (VL). Distributions were obtained from 10,000 simulations for each disease.

All the distributions of R_0_ look very similar (figure 1). They all are right-skewed distributions with, unsurprisingly, a majority of R_0_ values being lower than 1. However, all pathogens remain able to spread (R_0_>1) in 15–30% (and up to 55% for MAL) of cases following their incidental introduction. In addition, the tails of the distributions include large values of R_0_, suggesting a true potential for strong outbreaks for all these diseases. To identify the key processes determining the spread of pathogens in such sink vector populations, we then looked at the effect of the various parameters of the model on R_0_.

**Figure 2:**
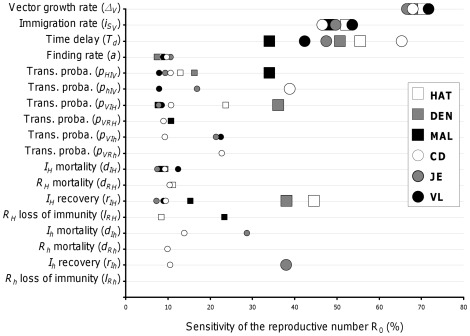
Sensitivity of the basic reproduction number (R_0_) to vector’s demography and feeding rates, and to pathogen’s transmissibility and virulence. All six vector-borne diseases appear on the same graph. Squares correspond to diseases with only human hosts: human African trypanosomiasis (HAT), dengue (DEN) and malaria (MAL). Circles correspond to diseases with non-human hosts: Chagas disease (CD), Japanese encephalitis (JE), and visceral leishmaniasis (VL). Larger symbols correspond to the key determinants of the variations of R_0_ (see main text for comments). Sensitivities were calculated from 10,000 simulations for each disease.

Our sensitivity analysis showed that vector-related parameters have the largest effects on R_0_ (figure 2). Demographic parameters, namely the local growth rate, representing the net balance between births, deaths and emigration (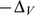), and the immigration (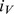) rate, are highly influential. Vector local growth rate has the largest effect because it determines both vector population abundance (which is equal to 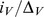), and the average time spent in the sink (which is equal to 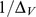), while immigration only has an effect on vector abundance. Variations in the time spent in the sink have an important impact on transmission, since they obviously influence the number of opportunities for vectors to encounter hosts. Vector feeding is another well-recognized factor in determining the rate of contact between vectors and hosts. Remarkably, by using the Antonovics et al.’s function of transmission [87], we were able to look at relative effect of the time delay between two blood-meals (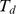), and the vectors finding rate (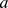). An interesting outcome is that the minimal amount of time between two blood-meals has a significant effect, similar to the impact of immigration, or even larger for the two trypanosomiases (HAT and CD). On the other hand, quite surprisingly, the vectors finding rate (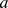) has virtually no impact on R_0_, whatever the disease being considered. This suggests that the spread of the pathogen is more limited by temporal constraints associated to the reproductive biology of the vector, than by its dispersal ability.

Parameters related to pathogen transmission and within-host dynamics typically have smaller and much more disease-specific effects. Still, the spread of DEN and HAT is significantly influenced by the human recovery rate (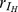). This is because at the typically low abundances encountered in sink vector populations, it is important that human hosts remain infectious for the pathogen to be transmitted back to the vectors. The spread of diseases with non-human hosts tends to be more sensitive to non-human hosts-related parameters, than to human hosts-related parameters. For similar reasons as explained above, the most important parameters are the rate of non-human hosts recovery and the probabilities of transmission between vectors and non-human hosts. Mostly, the non-human hosts recovery rate (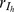) has a noticeable effect on the spread of JE, and the transmission probability from vectors to non-human hosts (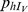) has an effect on CD. Finally, all the remaining parameters have lower effect, or virtually no impact on R_0_.

#### Prevalence of pathogens in sink vector populations

Results of the previous sections have clarified the conditions for the pathogens to spread in sink populations. While such spread relies only on vector immigration when pathogens are introduced via immigrating vectors (since R_0_ = 1+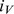), it is influenced by vector local growth rate (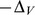), and the minimal amount of time between blood-meals (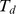), when pathogens are introduced independently of immigrating vectors. To determine if the same processes were also the key determinants of pathogen’s prevalence when it becomes established in the population, we looked at the distribution of the percentage of infectious and recovered humans obtained while randomly sampling into the range of plausible parameter values (Table 1).

**Figure 3:**
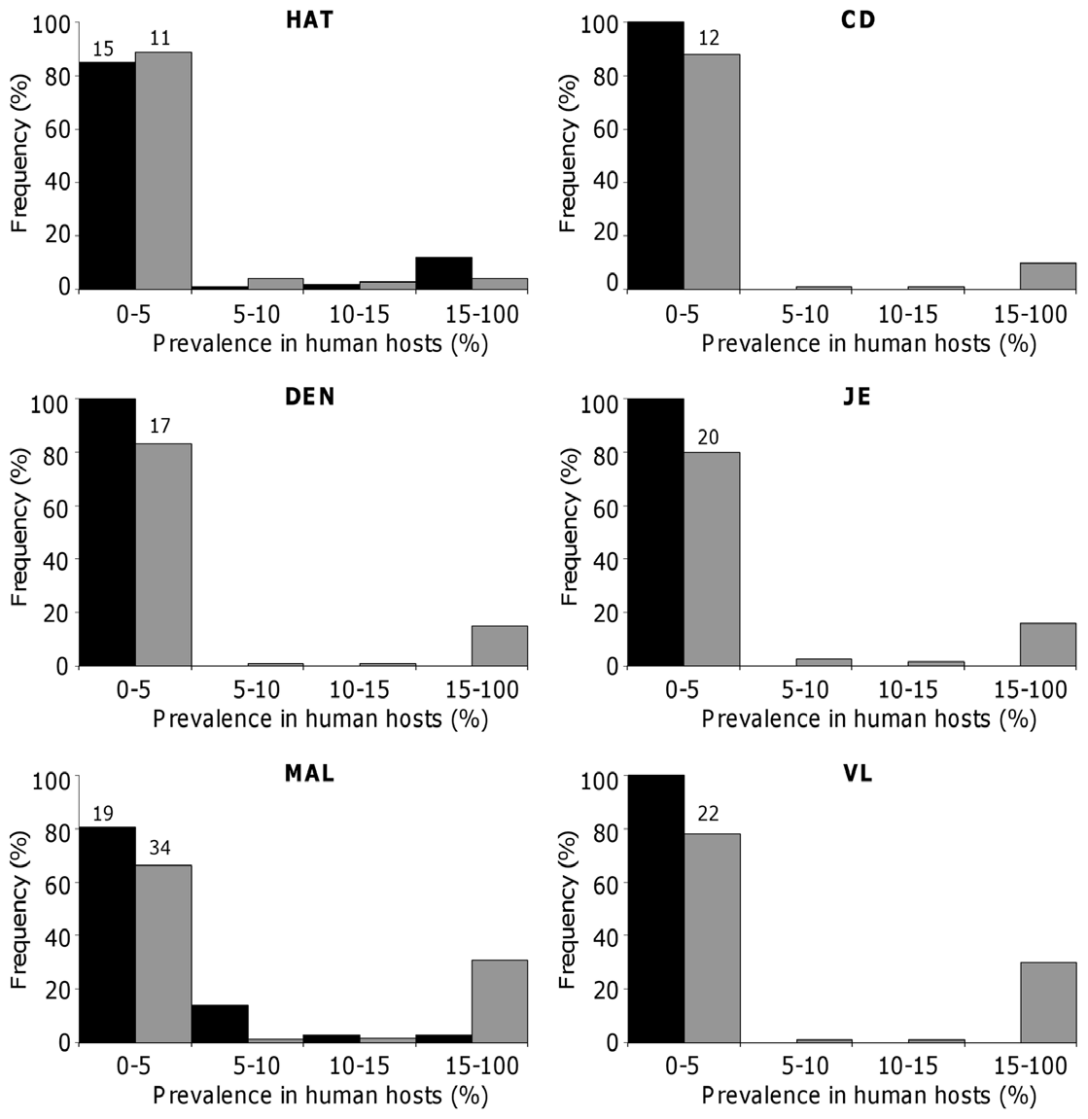
Distribution of the prevalence of infectious and recovered humans when no immigrant vector is infectious (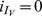). Black and grey bars give the prevalence of infectious (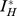) and recovered (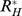) humans, respectively. Numbers above bars give (if any) the percentage of simulations leading to prevalence larger than 5%. Distributions were obtained from 10,000 simulations for each disease.

Independent introduction of vectors and pathogens in the sink vector population. The distribution of infection in humans shows that, when no immigrating vectors is infectious, the percentage of humans being infectious (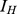) or ‘recovered’ (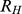) are lower than 5% in most conditions obtained from our random sampling (figure 3). For DEN, JE and VL, the percentage of infectious humans is systematically less than 5%, while the percentage of immune ‘recovered’ individuals can be more than 5% in roughly 20% of cases of pathogen’s introduction for each of these diseases. For MAL, the percentage of infectious humans can be significantly higher, since 19% of prevalence values are larger than 5%. Concomitantly, the percentage of immune ‘recovered’ individuals is also larger, with around 35% of the predicted prevalence larger than 15%. The higher prevalence of humans infectious with MAL is explained by a longer duration of infection (generated by lower rates of death and/or recovery of infected individuals) than for DEN, JE or VL. This, in turn, results in a higher prevalence of ‘recovered’ (and reduced infectivity) individuals in MAL than in these 3 other diseases. For HAT and CD, infectious and ‘recovered’ human hosts are both infected with the pathogen since they correspond to the two different phases of the disease. The percentage of infected human hosts (in either one or the other phase of the diseases) can, as for MAL, be larger than 5%. Typically, 10–15% of simulations lead to more than 15% of humans affected by HAT, and around 10% of simulations lead to more than 15% of individuals chronically infected with CD. Again, the higher rates of infection for these two trypanosomiases than for DEN, JE and VL, are mostly due to longer durations of infection, which result in larger accumulations of human cases despite low vector abundances. Overall, although all prevalence values are expectably lower than observed in typical vector source populations, ‘anthropic’ or naturally occurring sink vector populations can thus represent serious potential threats. If the pathogens is to be accidently introduced in such populations by the arrival of infected hosts, one expects 0–5% of the population to be affected by DEN, JE and VL, and even a larger fraction of the population to be suffering from diseases with longer duration of infection such as MAL, HAT and CD.

**Figure 4:**
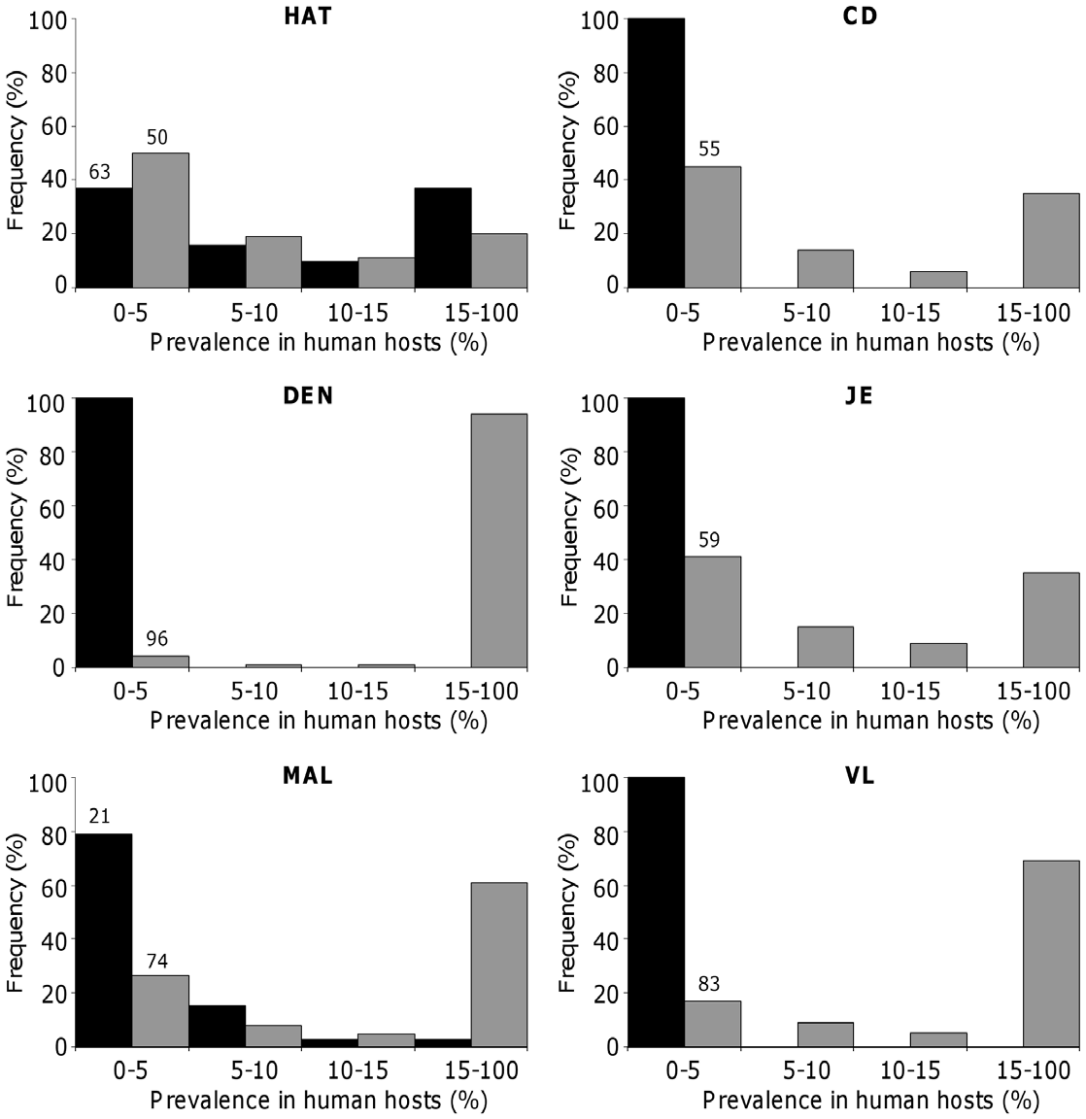
Distribution of the prevalence of infected and recovered humans when some immigrant vectors are infectious (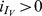). Black and grey bars give the prevalence of infectious (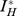) and recovered (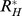) humans, respectively. Numbers above bars give (if any) the percentage of simulations leading to prevalence larger than 5%. Distributions were obtained from 10,000 simulations for each disease.

Introduction of pathogens via immigrating vectors. The distribution of prevalence in humans is modified when some immigrating vectors are infectious (figure 4). For DEN, JE, and VL, the percentage of infectious humans remains always lower than 5%. However, it is rather clear that the pathogen has infected many more individuals. The percentage of cases with more than 5% of immune ‘recovered’ individuals is indeed 3–6 times higher than when no immigrant is infectious (figure 3), and there is now more than 90%, more than 70% and 35% of simulations where more than 15% of individuals are immune to DEN, VL and JE, respectively. Similar changes were observed for MAL, though in smaller proportion. The percentage distribution of 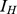 individuals remains virtually the same as when no immigrant is infectious (figure 3), but the transmission of the pathogen has also increased since the proportion of cases where more than 5% of individuals are ‘recovered’ raises from 34% to 74%. It is clear that transmission of HAT and CD was also much higher. For HAT, this manifested by a shift of the distribution of prevalence of the two stages of the diseases, with 4–5 more simulations where the prevalence of infectious and ‘recovered’ individuals were more than 5%. By contrast, for CD, only the prevalence of the second chronic phase of the disease markedly raised with 5–6 more simulations leading to more than 5% of chronically infected individuals. The difference between the two trypanosomiases is consistent with the much longer duration of the chronic stage than the acute phase of CD. Overall, the percentage of people currently suffering from DEN, JE, MAL, and VL, i.e. ‘infectious’ individuals, is not significantly higher when some immigrants are infectious, although the circulation of the pathogens in human hosts has been increased. This suggests that the within-host dynamics of the pathogen plays a critical role in determining the prevalence of infection for these diseases. On the contrary, the prevalence of individuals affected by HAT or CD, i.e. both ‘infectious’ and ‘recovered’ individuals, increased significantly when some immigrants are infectious. Such an increase for CD is clearly due to the high prevalence of infectious triatomines (resulting from their long life expectancy). For HAT, such an increase is rather explained by the very low probability of transmission from infectious humans to vectors, which strongly constrains the circulation of the pathogen. Compensating for this low probability, by introducing already infectious vectors, strongly facilitates the spread of the disease.

**Figure 5:**
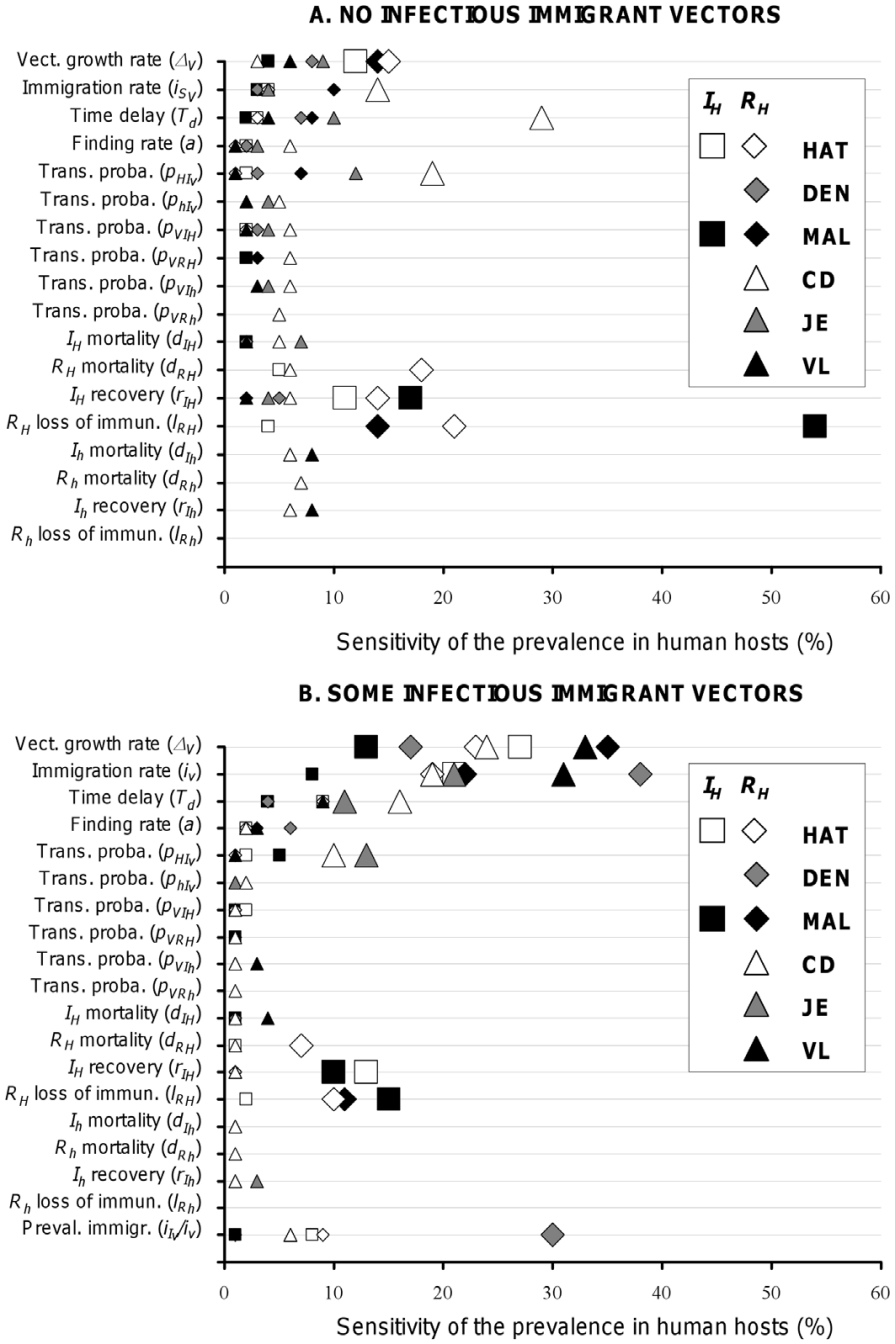
Sensitivity of the prevalence of infectious (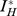) and ‘recovered’ (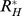) humans to vector’s demography and feeding rates, and to the pathogen’s transmission and within-host dynamics. (**A**) No immigrant vector is infectious (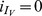). (**B**) Some immigrant vectors are infectious (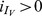). All six vector-borne diseases appear on each of the two graphs. Squares and diamonds correspond to the prevalence of infectious and recovered humans, respectively, for diseases with only human hosts: human African trypanosomiasis (HAT), dengue (DEN) and malaria (MAL). Circles and triangles correspond to the prevalence of infectious and recovered humans, respectively, for diseases with non-human hosts: Chagas disease (CD), Japanese encephalitis (JE), and visceral leishmaniasis (VL). Larger symbols correspond to the key determinants of the variations of prevalence in humans (see main text for comments). Sensitivities were calculated from 10,000 simulations for each disease.

To identify the key parameters determining those variations in the level of pathogen prevalence in humans, we performed a sensitivity analysis summarized in figure 5 (and figures S1 and S2). For DEN, JE, and VL we focused on the ‘recovered’ individuals since the prevalence of infectious individuals remains lower than 5% in all simulated conditions (see figures 3 and 4). Prevalence of ‘recovered’ provides a better picture of the overall transmission of pathogens to humans, especially because individuals in this category had long lasting immunity to DEN, JE or VL. We also focused on ‘recovered’ humans for CD since there are much more individuals in the chronic than in the acute stage of the disease. For MAL and HAT, both percentages of infectious and ‘recovered’ individuals reached higher levels, and we thus accounted for these two categories.

Sensitivity analysis for the independent introduction of vectors and pathogens. The vector-related parameters are no longer systematically the key parameters in determining the percentages of infectious or ‘recovered’ individuals (figure 5A and S1), as they were in influencing R_0_ (figure 2). The influence of vector- and pathogen-related parameters now varies from one disease to another. For DEN, VL and JE, there is no key parameter. The sensitivities of prevalence to each of the parameters were indeed roughly similar and lower than 10%. On the contrary, for the other three diseases, 2 to 4 parameters had marked effects exceeding 10%. The prevalence of infectious individuals with MAL was critically influenced by two parameters related to the within-host dynamics of the pathogen. First, the rate of recovery from infection (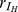), which determines how long individuals stay in the pool of highly infectious individuals. Second, the rate of return to a susceptible state (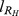), which directly influences both the pool of individuals that can be infected and the number of hosts from which the pathogen can be uploaded by vectors. On the contrary, vector-related parameters were the most influential on the percentage of individuals chronically infected with CD. These included, the minimal amount of time between two blood-meals (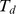) and immigration (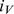), as well as the probability of transmission of the disease from vector to humans (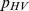), which all together determine the force of infection to humans. Interestingly, the analysis for HAT showed an intermediate pattern as key parameters were both vector- and within-host dynamics-related. Understandably, the human rate of return to the pool of susceptible (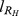) and the virulence to individuals in the second phase of the disease (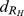) had a major impact on the loss, and thus on the prevalence of ‘recovered’ individuals. Similarly, the rate of transition to the second phase of the disease (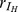) had a direct significant effect on the prevalence of individuals in the first phase of the diseases, i.e. ‘infectious’. However, the vector local growth rate (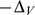) and the probability of transmission to humans (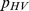) also had an impact on the prevalence of both ‘recovered’ and ‘infectious’ individuals.

Sensitivity analysis for the introduction of pathogens via immigrating vectors. When some immigrating vectors were infectious (see above), the key factors allowing for disease’s emergence and shaping the epidemiological dynamics that follows the initial spread of the pathogen could be identified from the sensitivity analysis of R_0_ (figure 2) and prevalence (figure 5A), respectively. The factors influencing the two stages of the dynamics can no longer be disentangled here since the pathogen spreads systematically. Accordingly, the parameters now influencing prevalence values (figure 5B and S2) are a combination of those that were shown to influence the R_0_ and prevalence in the previous situation. The most influential parameters are vector-related parameters (previously determining R_0_), eventually followed by additional parameters with smaller but noticeable effects. Interestingly, the latter are then the parameters that influenced prevalence when pathogens and immigrating vectors were introduced independently in the sink population. For all diseases, vector demography (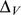 and 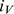) had the most influential effect, although the differences with the effect of other parameters were typically lower than what they were for R_0_ (figure 5B to be compared to figure 2). Only for individuals highly infectious with MAL (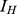), the rate of recovery (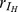) and the rate of return to the pool of susceptible (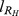) had a similar influence as vector demography (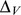 and 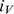). This is very consistent with the results obtained when no immigrating vector was infectious since the exact same parameters describing the within-host dynamics were already determining the prevalence of infection with MAL (figure 5A). Similarly, the parameters that were shown to influence the prevalence of HAT (i.e., 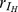, 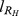 and 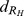) and CD (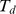 and 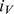) in the previous situation (figure 5A) are still playing a significant role in determining the rate of human infections (figure 5B). Finally, it is worth noting that the percentage of infectious vectors has low influence on human prevalence, except for DEN. This is mostly explained by the very low prevalence of infection in humans (figure 4) combined with the absence of non-human hosts. Opportunities for a susceptible vector to get infected are thus very limited, and can be substantially raised by the arrival of infectious immigrants, which makes the dynamics of the pathogen in the sink sensitive to the prevalence in dispersing vectors.

## Discussion

The concepts of ‘source’ and ‘sink’ have played a pivotal role in ecology by improving our understanding of species persistence out of their fundamental niche [27], [28], coexistence between competitive species (e.g., [98]) and predator-prey relationship (e.g., [99]). Such advances underline many decisions in today’s conservation biology (e.g., [100]). Surprisingly, those concepts have not been applied to improve our understanding of the transmission of human vector-borne diseases, and our ability to control such diseases, while many populations of transmitting vectors actually are ‘natural’ (e.g., [37], [70], [101], [72]) or ‘anthropic’ (typically generated by partially effective control intervention, [49], [50]–[54], [35], [58]) ‘sinks’. We aimed at identifying the key factors determining the possibility of emergence, and subsequent prevalence of infection, of six major human vector-borne diseases in such ‘sink’ populations. The approach intended was to design a unique ‘strategic model’ as a tool for qualitative and quantitative reasoning [102]. Such a ‘core’ model [65] does not allow accounting for disease specific processes, e.g. strong spatio-temporal heterogeneities or host feeding preference [88], which clearly are of fundamental importance to make predictions about the distribution and control of any particular pathogen [103]. The main results discussed below thus provide general insights that should now be contemplated and challenged by disease-specific models relying on detailed quantitative knowledge of particular systems.

### Emergence of Vector-borne Diseases in Sink Vector Populations

A first interesting outcome of our analyses is that all six human diseases were able to spread in about 15–30% (and up to 55% for MAL) of cases when pathogens are introduced accidently in a susceptible sink vector population, with potentially high reproductive ratio (R_0_) despite low vector abundance. The sensitivity analysis of R_0_ to the different parameters of the model showed that vector-related parameters (longevity, immigration, and feeding frequency) had the strongest influence on disease emergence. This pattern was very consistent across all six diseases, which suggests that it is a robust conclusion regardless of the existence of non-human hosts, and of the specificity of the transmission and within-host dynamics of the pathogens. More specifically, vector longevity is the key parameter in determining whether or not a pathogen would spread, and it has a larger effect on R_0_ than immigration and feeding frequency. Interestingly, while vector immigration (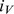) and longevity (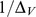) play a symmetrical role in determining vector abundance in a sink population since the latter is formally given by their product 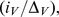 these two components bring different contributions to the emergence of pathogens in such populations. The rationale behind this differential sensitivity is quite simple and consistent with our understanding of factors influencing emergence in vector source populations (e.g., [61]). While different combinations of vector longevity and immigration can lead to identical vector abundance, the larger the longevity the smaller the turnover of the population. This, in turn, favours disease emergence since it requires vector individuals to live long enough to get infected and to infect a host back. To reinforce this conclusion, it is worth noting that the importance of vector longevity in the sink is undoubtedly underestimated here since we did not account for any development time of pathogen within the vector. Such delay would reduce the number of potentially infective contacts, and thus make the time spent in the sink population even more critical, especially for the emergence of diseases transmitted by short-lived vector such as DEN, MAL and JE. Similar effects of the interaction between the extrinsic incubation period and the survival rate of the vector have been demonstrated on the ground of general models [104], and more specific modelling of dengue [105] and malaria [106]. One must also point out that outbreak cycles of dengue are known to be influenced by the epidemiological status of the populations [107], so that, along with the above parameters, the transmission history in a given place is expected to have a strong influence on the spread of pathogens. The asymmetrical role of immigration and vector longevity has implications for pathogen transmission in a mosaic of vector habitats. One can indeed assume that as the distances or the ‘impermeability’ of the matrix between sources and sinks (e.g., [108]) increases, individuals reaching sinks will not only be fewer but also older, which will contribute (even more than the reduction in the number of individuals) to prevent the spread of the pathogen. Although this could be mitigated by the increase in the prevalence of infection with the age of the vector, the level of fragmentation of the landscape (i.e., many small and nearby patches instead of a few large and distant patches) is thus expected to favour disease emergence, not only because it increases the number of dispersers [109], [110], but also because it changes the age-structure of the immigrants. The differential effect of longevity and immigration may also be relevant in the context of ‘anthropic’ sinks if control had an impact on the age-structure of the immigrants. Indoor insecticide spraying is indeed known to induce dispersal of individuals receiving sub-lethal doses [111], or to select for exophilic individuals at the population scale [50]. If such effects were biased towards the youngest individuals, either because of an age-dependent behavioural response to chemicals, or because genotypes dispersing earlier in life would be selected for, the spread of the pathogen in surrounding sinks could then be favoured. Finally, given the importance of vector longevity, one would have expected HAT and CD to spread more easily than other diseases, since tsetse flies and triatomines have longer life-expectancy. On the contrary, the values of R_0_ were found very similar for all diseases. This implies that other disease specificities are balancing against the risk factors associated to vector life-history. Indeed, HAT and CD are both characterized by very low transmission probabilities between vectors and humans, which undoubtedly lowered the rates of spread of these two trypanosomiases. Thus, although vector life-history and feeding were critical to explain variations in pathogen’s reproductive rate for each of the diseases considered, they did not induce significant in-between diseases differences in the risk of emergence. Thus one cannot point out human vector-borne pathogens that would be more prone to emerge in vector sink populations. Vector sink populations appear to be a real threat of emergence or re-emergence of all six human vector-borne diseases considered here. As expected, vector control in the source will have an important effect on the rate of spread of the pathogen in the connected sink populations. Interestingly, control interventions in the source that would reduce vector longevity in the sink appear to be as relevant as interventions that would directly reduce the number of vector individuals migrating from the source into the sinks.

### Prevalence of Vector-borne Diseases in Sink Vector Populations

Our analyses show that even in a disease-free sink vector population (sustained by the immigration of non-infectious vectors), the spread of the pathogen (when introduced accidentally by infected hosts) can potentially represent significant health concern. Prevalence of infection larger than 5% is observed in up to 11–34% of cases for diseases with long duration of infection such as MAL, CD and HAT. In addition, when the prevalence of infection remains lower than 5%, such as for DEN, VL and JE, the pathogens actually spread through a more substantial part of the population since the percentage of ‘recovered’ individuals is larger than 5% in about 20% of cases. When pathogens are regularly introduced by immigrating vectors, the spread of the pathogens was expectably facilitated. However for DEN, VL and JE the prevalence did not significantly increase. This is mostly because vectors have a short life expectancy, so that the prevalence of infection hardly exceeds 2% among immigrants. In any case, the percentage of humans afflicted by any of the six diseases typically remains lower than 15%. These figures are consistent with the few estimates available from areas where vector populations are known or expected to be sinks. In the Yucatán peninsula, Mexico, sink populations of non-domiciliated triatomines [38], [112], [113] are responsible for human sero-prevalence rates of 5–18% [71]. Similarly, wild sandflies species are responsible for 2–3% prevalence of leishmaniasis (calculated from incidence in [114]), and transmission by sylvatic species of glossines leads to less than 5% of the Gambian form of sleeping sickness in West and Central Africa [115]. Finally, the prevalence of highly infectious individuals with MAL is consistent with the less than 10% of infection typically observed in areas where the transmission of the pathogen is associated with vector dispersal. Examples include dispersal in urban areas representing a fragmented habitat for *Anopheles*, or dispersal from sites located at lower or most suitable altitudes [116]–[118].

Prevalence values that could be reached if a pathogen was to be introduced in a sink population of susceptible vectors are overall influenced in a much more comparable way by vector’s (demography and feeding) and pathogen’s (transmission and within-host dynamics) parameters, than R_0_ was in the same epidemiological situation (see first part of the discussion). No important parameter could be identified for the transmission of DEN, VL and JE, and key parameters were disease specific for CD, HAT and MAL. For CD, prevalence was mostly determined by vector-related parameters, which is best explained by the strikingly low probability of ‘stercorarian’ transmission of the pathogen to mammals [101], [119]. On the contrary, the prevalence of humans suffering from MAL and HAT was mostly influenced by parameters related to the pathogen-humans interaction; rate of recovery, loss of immunity and disease-induced mortality, as it is usually the case when there are only human hosts for the pathogen [24]. However, when pathogens were introduced through vector immigration, the importance of vector longevity and immigration was again prominent, although the transmission and within-host parameters mentioned above still had some influence on prevalence. Overall, this confirms that vector demography and feeding rates are the key determinants of disease dynamics, apart for HAT and MAL for which variations in pathogen’s interaction with its human host also is influencing its prevalence.

Such a conclusion reinforces the idea that the key determinants of epidemiological dynamics are roughly similar for all the pathogens that we considered in sink vector populations. The primacy of vector-related parameters has implications for the control of transmission to humans. Essentially, reducing vector presence in human habitat could readily be efficient even if vector abundance is already typically low. In such situations, public health policies promoting drug administration should thus not undermine vector control programs. Clearly, control intervention in source populations are expected to have an impact on prevalence in the connected sink populations. Another implication of our results is that, even if human transmission is reduced through vector control programs in source populations, small residual level of infection in vectors can still be responsible for the spread of the pathogen in surrounding sink populations. This corroborates the previous conclusion that, when implementing control strategies, interruption of transmission should be targeted at larger scale rather than in areas of high transmission [120].

### Conclusion and Potential Guidelines for Field Studies

Our analyses indicate that sink vector populations can represent serious threats to human health, with 1–15% prevalence of key vector borne diseases. Such ‘residual’ transmission is expected to be especially noticeable for diseases with long duration of infection, such as the African and American trypanosomiasis, but also appears relevant to other diseases. Our results thus have potential implications for future theoretical and field studies of vector-borne diseases.

First, to understand pathogen transmission and evolution will require to account for sink vector populations (within a typical mosaic of vector habitats), and then to properly disentangle local growth from immigration since these two processes have different effects on the R_0_ and prevalence of the pathogens. Estimates of local vector abundance provided by population or genetic studies, which represent the combined outcome of local growth and immigration, will thus only be worth collecting if they provide enough information on spatial structures that allow inferring about local adaptation and immigration, possibly through an approach of model selection [113], [121]. Second, incomplete interruption of transmission in areas of high vector abundance will still allow for the pathogen to spread in surrounding sink populations, which implies that vector control programs should be considered a meta-population context [122], and implemented at larger scale than areas of high vector densities. Third, as pathogen transmission and within-host dynamics have low influence on disease dynamics, different strains are expected to spread similarly in sink vector populations and, accordingly, selection on virulence is expected to be weak in such habitat. Although evolution in a mosaic of source-sink habitats has been investigated for non-pathogenic species (e.g., [123]), it has been widely overlooked in studies of vector-borne pathogens. Our results suggest that considering a realistic source-sink dynamics for vector populations, may alter our conclusion on pathogen transmission by promoting strain diversity and affecting the evolution of virulence. A similar conclusion was recently reached about the plausible effect of temporal dispersal, arising from vector developmental delays, on the spread and prevalence of vector-borne pathogens [124].

Much theoretical and field study is needed on the ecological and evolutionary potential of sink vector populations if one is to frame more substantially the control of infectious diseases in the context of meta-population, as it has already been proved successful for conservation biology [125].

## Supporting Information

Figure S1
**Sensitivity analysis for the prevalence in humans when no immigrant vector is infectious.** The widths of arrows are set up according to the value of sensitivity appearing in figure 5A. Symbols correspond to the key-parameters identified in the main text, and are set next to processes (arrows) in which they are involved. For each disease, the compartments of interest are represented as in figure 5A (e.g., black square and diamond for MAL 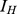 and 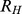 individuals, respectively), while all other compartments are round-shaped (e.g., MAL susceptible individuals). For MAL and HAT, full and dashed arrows refer to the influence of parameters on the prevalence of ‘recovered’ and infectious human hosts, respectively.(TIF)

Figure S2 **Sensitivity analysis for the prevalence in humans when some immigrant vectors are infectious.** The legend is the same as for figure S1, though values of sensitivity and key parameters now appear as identified in figure 5B rather than figure 5A.
(TIF)

Text S1 **Estimates of parameters.**
(DOC)
