# Supplementary figures and images for: Emergence and Prevalence of Human Vector-Borne Diseases in Sink Vector Populations
Source: PLoS One. 2012 May 18;7(5):e36858. doi: 10.1371/journal.pone.0036858 (PMC3356347; doi:10.1371/journal.pone.0036858)

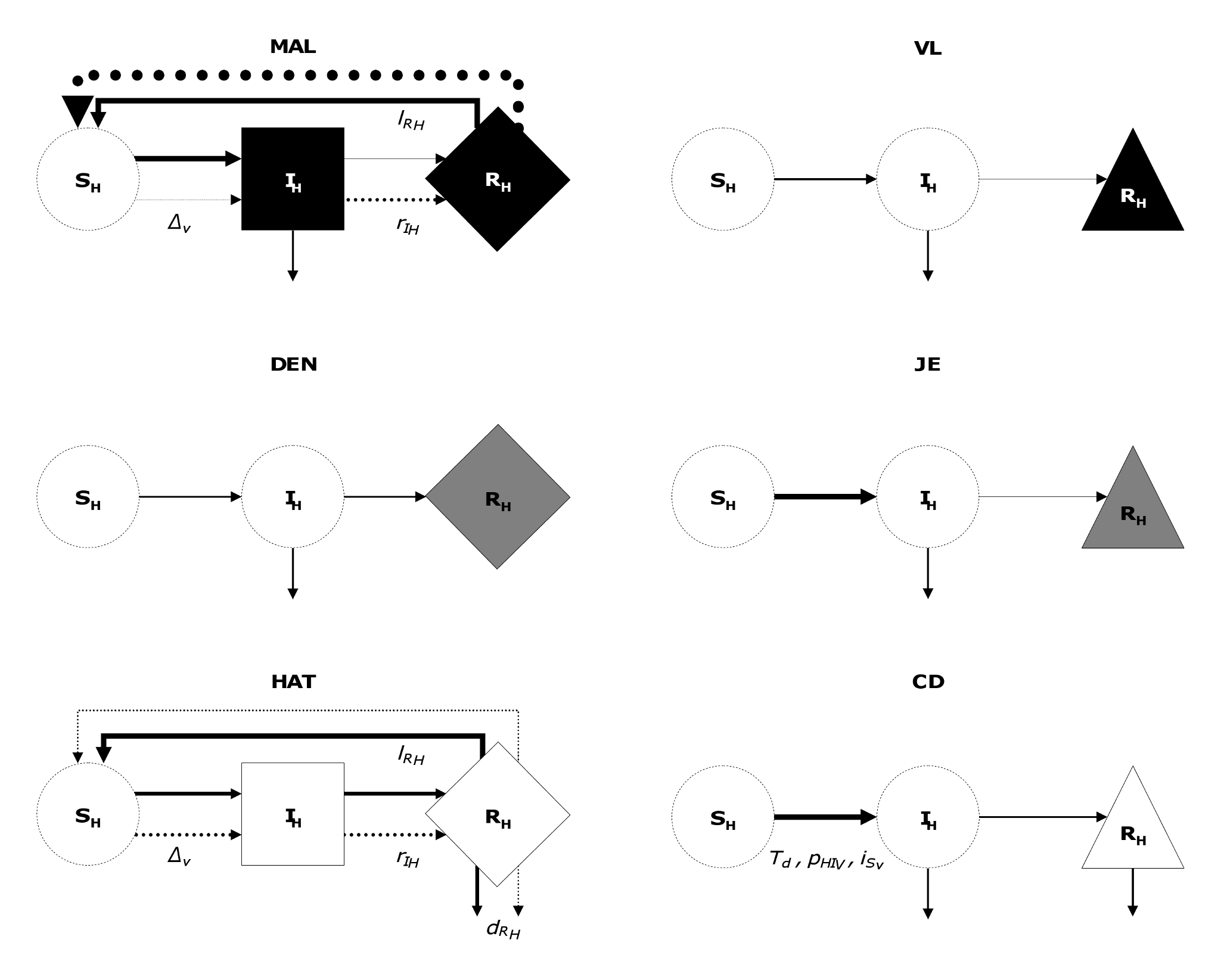

Supplement: Figure S1 — Sensitivity analysis for the prevalence in humans when no immigrant vector is infectious. The widths of arrows are set up according to the value of sensitivity appearing in figure 5A. Symbols correspond to the key-parameters identified in the main text, and are set next to processes (arrows) in which they are involved. For each disease, the compartments of interest are represented as in figure 5A (e.g., black square and diamond for MAL and individuals, respectively), while all other compartments are round-shaped (e.g., MAL susceptible individuals). For MAL and HAT, full and dashed arrows refer to the influence of parameters on the prevalence of ‘recovered’ and infectious human hosts, respectively. (TIF) [file pone.0036858.s001.tif]

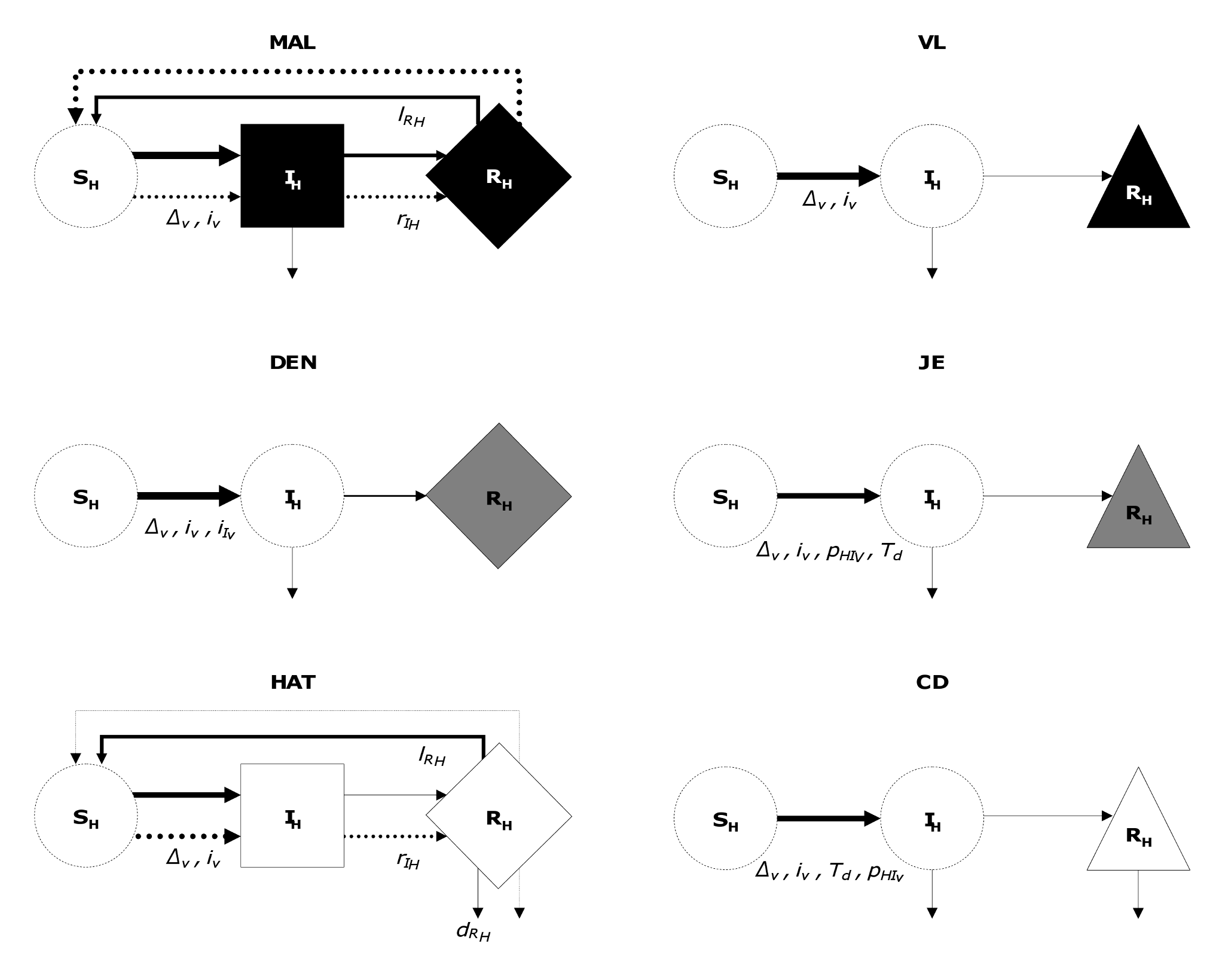

Supplement: Figure S2 — Sensitivity analysis for the prevalence in humans when some immigrant vectors are infectious. The legend is the same as for figure S1, though values of sensitivity and key parameters now appear as identified in figure 5B rather than figure 5A. (TIF) [file pone.0036858.s002.tif]
