# Supplementary material for: Emergence and Prevalence of Human Vector-Borne Diseases in Sink Vector Populations
Source: PLoS One. 2012 May 18;7(5):e36858. doi: 10.1371/journal.pone.0036858 (PMC3356347; doi:10.1371/journal.pone.0036858)
Supplement: Text S1 — Estimates of parameters. (DOC) [file pone.0036858.s003.doc]

**Text S1: Estimates of Parameters**

This appendix provides a review of the estimates of the parameters defined in table 1, that we have been able to gather from the literature. Most papers we are referring to are empirical research papers with data from experimental lab work or field studies.

Demography and feeding rates of the vectors

*Vector local growth rate ($\Delta_{V}$).* The range of variation in vector growth rate was determined according to variations in vector longevity, neglecting the typically low reproduction of maladapted vectors in sink populations (see, e.g., [1]). Since the modelled vectors are thought to live shorter in sink populations, we used the values of longevity typically estimated in source populations (Table S1), as a clue of the maximum vector life expectancy in the sink. The minimal value of vector life expectancy in the village was set to one day. The range of $\Delta_{V}$ values to be used was then calculated assuming $\Delta_{V}$=1/vector life expectancy.

**Table S1.** Vectors adult longevity.

| **Vector (Disease)** | **Estimates (days)** | **Reference** |
| --- | --- | --- |
| Mosquitoes (MAL – DEN) | 5.8 - 20 | [2] |
| Mosquitoes (JE) | 2 - 29 | [3] |
| Sandflies (VL) | 6 - 27 | [4] |
| Tsetse flies (HAT) | 45 (20 - 60) | [5] |
| Triatomines (CD) | 210 (102 - 338) | [6,7] |

Because the estimates found in the literature for mosquitoes (transmitting MAL, DEN and JE) and sandflies (transmitting VL), were roughly similar, they were given the same standard value of 15 days corresponding to the median of their ranges. According to the other estimates collected, tsetse flies (transmitting HAT) were considered to live up to 45 days (the median of the observed values), while the longevity of triatomines (transmitting CD) was fixed to 210 days (the median of the observed values).

*Vector immigration ().* The maximal rate of vector immigration was set for the ratio of vector to human densities to be ≤ 1. Since the total number of vectors at equilibrium equals , the maximal immigration rate was given by with , that is $\Delta_{V}N_{H}$. Using the maximal values of $\Delta_{V}$ specified above and the estimate of the number of human hosts $N_{H}$ (see below), the maximal daily rate of immigration was found to be approximately 67 individuals for mosquitoes (transmitting MAL, DEN and JE) and sandflies (transmitting VL), 22 individuals for tsetse flies (transmitting HAT), and 5 for triatomines (transmitting CD). The minimal rate of immigration was set to 0 immigrants per day for all vectors.

*Prevalence of infection in immigrant vectors ($i_{I_{V}}/i_{V}$)*. Prevalence of infection is typically low in mosquitoes (e.g., 2.1% in *Anopheles* - MAL, [8], 0.097% in *Aedes* - DEN, [9], and 1.67% in *Culex* - JE, [10] and flies (0.18% in *Glossina* - HAT, [11], and 1.56% in phlebotomines - VL, [12]). We thus let the prevalence in immigrant vectors of MAL, DEN, JE, VL, and HAT takes on small values within a common range: 0 to 2%. Levels of prevalence of *Trypanosoma cruzi* found in triatomines are much higher, typically of the order of 20-50% (e.g., [13-17]), and we thus used an upper limit of 35% that corresponds to the median of the observed values.

*Minimal amount of time between two blood-meals ($T_{d}$).* The length of the gonotrophic cycle is commonly thought to be the main determinant of the time elapsed between 2 consecutive contacts made by a vector individual with its host. For dipterous species, such as of mosquitoes or flies, blood-feeding on vertebrate/human hosts, this time interval is on average 3-4 days (see e.g., [2] for *Anopheles*, [18] for *Glossina*, [19] for phlebotomines). We thus let parameter $T_{d}$ vary in a typical range of 2 to 6 days for the five diseases transmitted by dipterous insects: MAL, DEN, JE, VL, and HAT. However, for the triatomines vectors of CD, this amount of time is significantly larger and have been shown to last from 6 days [20] to 13-26 days [21]. Accordingly, the minimal amount of time between two blood-meals for CD transmitting bugs was varied between 1 and 4 weeks.

*Vector finding rate ($a$).* There is virtually no estimate of vector finding rate in the literature (but see [22]). We thus choose to set up the limits of parameter $a$ to allow for a daily rate ranging from 0 to 100% of the modelled human population.

Demography of human and non-human hosts

*Human and non-human hosts population size ($N_{H}$, $N_{h}$).* Human population size ($N_{H}$) was set up to 1000 individuals to model a standard village or a small town. Key domestic non-human hosts of the diseases considered in this study are dogs (for CD and VL) and swine (for JE). [23] recently reviewed dog populations in 23 locations over 14 developing countries. The number of dogs per hundred inhabitants in rural areas typically ranged from 9 to 33.6, with an average of 16.9. A similar estimate could be derived from a study of domestic swine in rural provinces of Lao [24]; the number of swine per hundred inhabitants there varied from 9.5 to 197.3. The corresponding ratio of dogs or swine to humans equals 5.9 (95% IC: 4.9-7.5) and 5.2 (95% IC: 4.0-6.3), respectively. In our model, this ratio was then set up to 1/6, and the number of non-human hosts ($N_{h}$) calculated accordingly, $N_{h}=1000/6\approx167$.

*Human and non-human hosts natural death rates ($d_{H}$, $d_{h}$).* Natural mortality rates were calculated as 1/longevity. They constitute the mortality rate of susceptible human and non-human hosts, and the baseline to which a disease-induced mortality (see ‘*Human and non-human mortality induced by the pathogen*’) was added to obtain the mortality rate of infectious and recovered human and non-human hosts. Human hosts natural life expectancy was set to 60 years. The longevity of domestic swine (transmitting JE) was set to 12 months, considering that animals are raised for humans meat-consumption and slaughtered at that age. The life expectancy of dogs, non-human hosts of VL and CD, was set to 3 years, in agreement with [23] and [25] who found that dogs’ longevity ranges from around 2.5 to 4 years.

Probabilities of transmission of the pathogens

*Transmission probability from an infectious* *vector to a human host ($p_{\text{HI}_{V}}$).* Estimates of those probabilities appear in table S2. As we could not find any estimate of the probability of transmission from an infectious sandfly to a human host, we used the estimate of the probability of transmission from a sandfly to a dog (see ‘*Transmission probability from an infectious* *vector to a non-human host’*).

**Table S2.** Probability of transmission from vector to human host.

| **Vector (Disease)** | **Range** | **Reference** |
| --- | --- | --- |
| Mosquitoes (MAL) | 0.01 - 0.13 | [2] |
| Mosquitoes (DEN) | 0.5 - 1.0 | [26,27] |
| Mosquitoes (JE) | 0.01 - 0.04 | [28] |
| Sandflies (VL) | 0.2 - 0.4 | same values as for dogs (see text) |
| Tsetse flies (HAT) | 0.5 – 0.7 | [29] |
| Triatomines (CD) | 0.6e-3 - 3.8e-3 | [30, 31] |

*Transmission probability from an infectious* *vector to a non-human host ($p_{\text{hI}_{V}}$).* The only value of the probability of transmission of VL from vector to non-human hosts that we found came from an earlier model of VL in dogs [32]. This probability was equal to 0.32 per bite, and we thus considered values in the range 0.2 to 0.4 per bite. For JE, we used estimates of the ability of mosquitoes (*Aedes albopictus*) to transmit the virus to susceptible mice, which ranged from 0.27 to 0.45 per bite [33]. Finally, we assumed vector transmission of CD to dogs to be equivalent to vector transmission to humans (see Table S2.).

*Probability of transmission from infectious or recovered humans to vector (*$p_{\text{VI}_{H}}$, $p_{\text{VR}_{H}}$*).* Values of the probability of transmission from infectious humans to vector we used appear in table S3. While estimates of this probability were available for MAL and DEN, we used estimates obtained from other mammals, that is hamster, mice and dog, for JE, VL, and CD, respectively (see ‘*Probability of transmission from infectious or recovered non-human hosts to vector*’). *Glossina* female flies (transmitting HAT) are considered to be susceptible only while taking their first blood-meal. The susceptibility of such ‘teneral’ females to *Trypanosoma brucei gambiense* ranges from 0.05 to 0.14 per bite [34,35]. We did not explicitly model this age-dependent susceptibility in tsetse (as, e.g., in [36]), but weighted the infectiousness of humans in early phase of HAT by the probability for a biting tsetse fly to be a ‘teneral’ individual. Such probability was estimated by considering that ‘teneral’ individuals have their first blood meal on their first day of life. Assuming a constant natural death rate of 1/45 per day (see table S1), and considering a stable age-structure, this probability (that equals the fraction of 1-day old individual in the population) was found to be equal to 0.034.

**Table S3.** Probability of transmission from infectious humans to vector.

| **Vector (Disease)** | **Range** | **Reference** |
| --- | --- | --- |
| Mosquitoes (MAL) | 0.24 - 0.64 | [2] |
| Mosquitoes (DEN) | 0.15 - 0.73 | [37] |
| Mosquitoes (JE) | 0.14 - 0.38 | [38] |
| Sandflies (VL) | 0.21 - 0.29 | [39] |
| Tsetse flies (HAT) | [0.05 - 0.14] * 0.034 | to limit susceptibility to teneral flies (see text) |
| Triatomines (CD) | 0.90 - 0.94 (0.99) | same values as for dogs (see text) |

Recovered humans were assumed to have cleared the pathogen for DEN, JE and VL (see section ‘Modelling’ in the main text), so that their ability to transmit to vector was set to 0. While modelling HAT, ‘recovered’ individuals are in the second stage of the disease. Though circulating pathogens could potentially be transmitted to vectors, it is commonly assumed to be unlikely because of the typically low pathogen concentration in the blood [40]. We thus set the probability of transmission from humans in the second stage of the disease to vector to 0. On the contrary, infectiousness of MAL ‘recovered’ individuals (which are thought to be able to transmit but with reduced infectiousness), and CD ‘recovered’ individuals (which are thought to be in the chronic phase of the disease), was considered positive. This infectiousness $p_{\text{VR}_{H}}$ was considered to range from 0.024 to 0.064 for MAL, that is, a probability ten times as small as $p_{\text{VI}_{H}}$, following [41], and from 4.2e-3 to 6.2e-3 for CD, following [42].

*Probability of transmission from infectious or recovered non-human hosts to vector (*$p_{\text{VI}_{h}}$, $p_{\text{VR}_{h}}$*).* Estimates of the probability of transmission from infectious non-human hosts to vector appear in table S4. Since estimates for CD were obtained on bugs’ larvae, and because adults are thought to be even more susceptible [42], the maximal transmission probability was increased from 0.94 to 0.99.

**Table S4.** Probability of transmission from infectious non-human hosts to vector.

| **Vector (Disease)** | **Range** | **Reference** |
| --- | --- | --- |
| Mosquitoes (JE) | 0.55 - 1.00 | [43] |
| Sandflies (VL) | 0.05 - 0.28 | [44] |
| Triatomines (CD) | 0.90 - 0.94 (0.99) | [45] |

Because we assumed that no dog recovered from VL, and that swine recovered from JE have cleared the pathogen (see section ‘Modelling’ in the main text), the only ‘recovered’ non-human individuals able to transmit pathogens to susceptible vectors were dogs chronically infected with CD. We then considered probabilities of transmission ranging from 0.05 [45] to 0.31 [42].

Within-host dynamics of the pathogens

*Human and non-human mortality induced by the pathogen (*$v_{I_{H}}$, $d_{I_{H}}$, $v_{R_{H}}$, $d_{R_{H}}$, $v_{I_{h}}$, $d_{I_{h}}$, $v_{R_{h}}$, $d_{R_{h}}$*).* The virulence of the different pathogens to infectious or ‘recovered’ human hosts was calculated from fatality rates collected in the literature (Table S5). The additional mortality induced by the pathogen was calculated as , where $T$ stands for the period of time over which the fatality rate was reported. $T$ was 365 days for MAL, since this disease fatality rate was evaluated on an annual basis. $T$ was set to the duration of the infectious stage (i.e., $1/r_{I_{H}}$) for all other diseases, since their fatality rates were evaluated per case.

**Table S5.** Rate of fatality for infectious humans and pathogen-induced mortality.

| **Disease** | **Fatality rate** | **Reference** | **Pathogen-induced mortality ($v_{I_{H}}$)** |
| --- | --- | --- | --- |
| MAL | 0% - 15% per year | [2] | 0 – 4.5e-4 per day |
| DEN | 0% - 2% per case | [46] | 0 – 6.7e-4 per day |
| JE | 5% to 40% per case | [47] | 3.7e-3 – 0.26 per day |
| VL | 4% - 98% per case | [48,49] | 2.3e-4 – 4.4e-2 per day |
| HAT | 0% per case | [50] | 0 |
| CD | 0% - 5% per case | [51] | 0 – 1.1e-3 per day |

We further considered an additional mortality for ‘recovered’ humans. Individuals in ‘recovered’ stage of the African and American trypanosomiasis, though they are no longer in the pool of infectious, are still at risk of death because they are in a second phase of the disease. For HAT, the fatality rate in the late phase of the disease can drop down to 2% if people are given drugs [52], while it can potentially reach 100% in absence of such treatment. Accordingly, the range of pathogen-induced mortality was set to 2.7e-5 - 3.8e-2 per day. For CD, up to one third of individuals in the chronic phase of the disease can die [51]. We thus varied the fatality rate from 0% to 33%, and the pathogen induced mortality from 0 to 1.8e-5 per day.

The virulence of JE, VL, and CD’s pathogens to infectious or ‘recovered’ non-human hosts were calculated from fatality rates in the same way as for virulence to human hosts. Fatality rates of infected swine that are non-human hosts of JE, can vary from 0 in adults to 100% in new-borns [53,54]. We considered this whole range of variation so that the additional mortality due to the diseases was varied from 0 to 4.61. Infected dogs that are non-human hosts of VL are typically killed through culling program. The fatality rate of infectious dogs was set to 99%, assuming that a small fraction of infected dogs was not killed because of inefficient detection, failed diagnosis, or non-participation of dog-owners [55,56]. The additional mortality due to the pathogen was then varied from 4.2e-3 to 4.61 per day. We could not find estimates of the fatality rate for infected dogs hosts of CD. We then assumed the fatality rates in both the acute ($I_{h}$) and chronic ($R_{h}$) stage of CD to be the same as in humans. Dog is indeed viewed as the best experimental model for studying CD pathology, because the course of the disease is very similar to what is observed in human hosts [57]. Accordingly, additional mortality induced by the pathogen was varied from 0 – 1.1e-3 per day in the acute stage, and from 0 – 3.6e-4 per day in the chronic stage of the disease.

*Human recovery and loss of immunity (*$r_{I_{H}}$, $l_{R_{H}}$*).* The rates of human recovery and loss of immunity were calculated as the inverse of the duration of infectious and ‘recovered’ stages, i.e. $1/r_{I_{H}}$ and $1/l_{R_{H}}$, respectively. Duration of the infectious state in human hosts used for the calculation of the recovery rates are reported in table S6.

**Table S6.** Duration of the infectious state in human hosts $1/r_{I_{H}}$.

| **Disease** | $1/r_{I_{H}}$ **(days)** | **Reference** |
| --- | --- | --- |
| MAL | 60 – 630 | [2] |
| DEN | 3 - 15 | [58,59] |
| JE | 2 - 14 | [60] |
| VL | 90 - 180 | [61] |
| HAT | 120 – 780 | [36,62] |
| CD | 45 - 60 | [63] |

For MAL, the rate of return to a susceptible and non-infectious state was calculated for the duration of reduced infectivity to range from 3 months to life-long, following [2]. For HAT, the rate at which individuals leave the pool of ‘recovered’ was calculated from the average duration of the late phase of the disease (state $R_{H}$), which ranges from 4 [36] to 24 months [62]. We assumed long-life immunity for JE, and VL, and considered that CD chronic infection also lasts for life.

*Non-human recovery and loss of immunity (*$r_{I_{h}}$, $l_{R_{h}}$*).* As for human hosts, the rates of recovery and loss of immunity of non-human hosts were calculated as the 1/duration of the $I_{h}$ and $R_{h}$ stages, respectively. We varied the duration of infection in swine hosts of JE from 1 day [64] to 7 days [65], and assumed long-life immunity for ‘recovered’ individuals. For VL, we considered a dog population where infection can be cleared by natural death or by cull-and-replacement program, as typically done in other modelling attempts, [55,66]. We let the possibility for the infection to be life-long in absence of control program, and reduced it to a unique day to mimic extremely timely interventions. Additionally, the rate of return to the susceptible stage was assumed to be infinitely large since in both cases, natural death or cull-and-replacement, dogs die (rather than recover) and are then typically replaced by a susceptible individual. In agreement with [57], we varied the duration of the acute phase of CD in dogs from 45 to 75 days, and assumed a life-long chronic stage of the disease, i.e. no return to the susceptible stage.

**REFERENCES**

1. Gourbière S, Dumonteil E, Rabinovich JE, Minkoue R, Menu F (2008) Demographic and dispersal constraints for domestic infestation by non-domiciliated Chagas disease vectors in the Yucatán peninsula, Mexico. Am J Trop Med Hyg 78: 133-139.

2. Chitnis N, Hyman JM, Cushing JM (2008) Determining important parameters in the spread of malaria through the sensitivity analysis of a mathematical model. Bull Math Biol 70: 1272-1296.

3. Fouque F, Carinci R, Gaborit P, Issaly J, Bicout DJ, et al. (2006) *Aedes aegypti* survival and dengue transmission patterns in French Guiana. J Vector Ecol 31: 390-399.

4. Srinivasan R, Panicker KN (1993) Laboratory observations on the biology of the phlebotomid sandfly, *Phlebotomus papatasi* (Scopoli, 1786). Southeast Asian J Trop Med Public Health 24: 536-539.

5. Gouteux JP, Laveissiere C (1982) Ecologie des glossines en secteur pré-forestier de Côte d’Ivoire 4. Dynamique de l’écodistribution en terroir villageois (1) Cah ORSTOM, sér Ent Med Parasitol 20: 199-229.

6. Rabinovich JE (1972) Vital statistics of Triatominae (Hemiptera: Reduviidae) under laboratory conditions. I. *Triatoma infestans* Klug. J Med Entomol 9: 351-370.

7. Zeledόn R, Cordero M, Marroquίn R, Lorosa ES (2010) Life cycle of *Triatoma ryckmani* (Hemiptera: Reduviidae) in the laboratory, feeding patterns in nature and experimental infection with *Trypanosoma cruzi*, Mem Inst Oswaldo Cruz 105: 99-102.

8. Atangana J, Bigoga JD, Patchoké S, Ndjemaï MN, Tabue RN, et al. (2010) Anopheline fauna and malaria transmission in four ecologically distinct zones in Cameroon. Acta Trop 115: 131-136.

9. Chen CF, Shu PY, Teng HJ, Su CL, Wu JW, et al. (2010) Screening of dengue virus in field-caught *Aedes aegypti* and *Aedes albopictus* (Diptera: Culicidae) by one-step SYBR green-based reverse transcriptase-polymerase chain reaction assay during 2004-2007 in Southern Taiwan. Vector Borne Zoonotic Dis 10: 1017-1025.

10. Jeong YE, Jeon MJ, Cho JE, Han MG, Choi HJ, et al. (2010) Development and field evaluation of a nested RT-PCR kit for detecting Japanese encephalitis virus in mosquitoes. J Virol Methods 171: 248-252.

11. Dagnogo M, Traoré G, Souleymane F (2004) Determination of sleeping sickness transmission risk areas from trypanosome infection rates of tsetse flies in Daloa, Côte d’Ivoire. Int J Trop Insect Science 24: 170-176.

12. Felipe IM, de Aquino DM, Kuppinger O, Santos MD, Rangel ME, et al. (2011) Leishmania infection in humans, dogs and sandflies in a visceral leishmaniasis endemic area in Maranhão, Brazil. Mem Inst Osw Cruz 106: 207-211.

13. Gürtler RE, Cohen JE, Cecere MC, Lauricella MA, Chuit R, et al. (1998) Influence of humans and domestic animals on the household prevalence of *Trypanosoma cruzi* in *Triatoma infestans* populations in northwest Argentina. Am J Trop Med Hyg 58: 748-758.

14. Dumonteil E, Gourbière S, Barrera-Pérez M, Rodriguez-Félix E, Ruiz-Piña H, et al. (2002) Geographic distribution of *Triatoma dimidiata* and transmission dynamics of *Trypanosoma cruzi* in the Yucatán peninsula of Mexico. Am J Trop Med Hyg 67: 176-183.

15. Dumonteil E, Gourbière S (2004) Predicting *Triatoma dimidiata* abundance and infection rate: a risk map for natural transmission of Chagas disease in the Yucatán peninsula of Mexico. Am J Trop Med Hyg 70: 514-519.

16. Ramirez-Sierra MJ, Herrera-Aguilar M, Gourbière S, Dumonteil E (2010) Patterns of house infestation dynamics by non-domiciliated *Triatoma dimidiata* reveal a spatial gradient of infestation in rural villages and potential insect manipulation by *Trypanosoma cruzi*. Trop Med Health 15: 77-86.

17. Nouvellet P, Ramirez-Sierra MJ, Dumonteil E, Gourbière S (2011) Effects of genetic factors and infection status on wing morphology of *Triatoma dimidiata* species complex in the Yucatán peninsula, Mexico. Infect Genet Evol 11: 1243-1249.

18. Rogers D (1977) Study of a natural population of *Glossina fuscipes fuscipes* Newstead and a model of fly movement. J Anim Ecol 46: 309-330.

19. Dye C, Davies CR, Lainson R (1991) Communication among phlebotomine sandflies: a field study of domesticated *Lutzomyia longipalpis* populations in Amazonian Brazil. Animal Behaviour 42: 183-192.

20. Rabinovich JE, Leal JA, Feliciangeli de Piñero D (1979) Domiciliary biting frequency and blood ingestion of the Chagas’s disease vector *Rhodnius prolixus* Ståhl (Hemiptera: Reduviidae), in Venezuela. Trans R Soc Trop Med Hyg 73: 272-283.

21. Schilman PE, Lazzari CR (2004) Temperature preference in *Rhodnius prolixus*, effects and possible consequences. Acta Trop 90: 115-122.

22. Botto-Mahan C, Cattan PE, Canals M, Acuña M (2005) Seasonal variation in the home range and host availability of the blood-sucking insect *Mepraia spinolai* in wild environment. Acta trop 95: 160-163.

23. Jackman J, Rowan A (2007) Free-roaming dogs in developing countries: the public health and animal welfare benefits of capture, neuter, and return programs. In: State of the animals. Salem D, Rowan A, editors. Human Society Press, Washington, DC. pp. 55-78.

24. Stür W, Gray D, Bastin G (2002) Review of the livestock sector in the Lao People’s Democratic Republic. International Livestock Research Institute. 57 p.

25. Reece JF, Chawla SK, Hiby EF, Hiby LR (2008) Fecundity and longevity of roaming dogs in Jaipur, India. BMC Vet Res 4:6.

26. Bartley LM, Donnelly CA, Garnett GP (2002) The seasonal pattern of dengue in endemic areas: mathematical models of mechanisms. Trans R Soc Trop Med Hyg 96: 387-397.

27. Focks DA, Daniels E, Haile DG, Keesling JE (1995) A simulation model of the epidemiology of urban dengue fever: literature analysis, model development, preliminary validation, and samples of simulation results. Am J Trop Med Hyg 53: 489-506.

28. Grossman RA, Edelman R, Chiewanich P, Voodhikul P, Siriwan C (1973) Study of Japanese encephalitis virus in Chiangmai valley, Thailand. II. Human clinical infections. Am J Epidemiol 98: 1211-1232.

29. Rogers DJ (1988) A general model for the African trypanosomiases. Parasitology 97: 193-212.

30. Rabinovich JE, Wisnivesky-Colli C, Solarz ND, Gürtler RE (1990) Probability of transmission of Chagas disease by *Triatoma infestans* (Hemiptera: Reduviidae) in an endemic area of Santiago del Estero, Argentina. Bull World Health Org 68: 737-746.

31. Nouvellet P, Dumonteil E, Gourbière S (2011) Estimating the unobservable: the ‘milli-transmission’ of Chagas disease to human. *In preparation*

32. Reithinger R, Colemen PG, Alexander B, Vieira EP, Assis G, et al. (2004) Are insecticide-impregnated dog collars a feasible alternative to dog cullings as a strategy for controlling canine visceral leishmaniasis in Brazil ? Int J Parasitol 34: 55-62.

33. Weng MH, Lien JC, Wang YM, Wu HL, Chin C (1997) Susceptibility of three laboratory strains of *Aedes albopictus* (Diptera: Culicidae) to Japanese encephalitis virus from Taiwan. J Med Entomol 34: 745-747.

34. Le Ray D (1989) Vector susceptibility to African trypanosomes. Ann Soc Belg Med Trop 69 Suppl 1: 165-171; discussion 212-214.

35. Artzrouni M, Gouteux J-P (1996) A compartmental model of sleeping sickness in central Africa. J Biol Syst 4: 459-477.

36. Artzrouni M, Gouteux J-P (2001) A model of Gambian sleeping sickness with open vector populations. IMA J Math Appl Med Biol 8: 99-117.

37. Hanley KA, Nelson JT, Schirtzinger EE, Whitehead SS, Hanson CT (2008) Superior infectivity for mosquito vectors contributes to competitive displacement among strains of dengue virus. BMC Ecol 8:1.

38. Doi R, Shirasaka A, Sasa M, Ova A (1977) Studies on the susceptibility of three species of mosquitoes to Japanese encephalitis virus. J Med Entomol 13: 591-594.

39. Vivenes A, Oviedo M, Márquez JC, Montoya-Lerma J (2001) Effects of a second bloodmeal on the oesophagus colonization by *Leishmania Mexicana* complex in *Lutzomyia evansi* (Diptera: Psychodidae). Mem Inst Oswaldo Cruz 96: 281-283.

40. Kennedy PGE (2004) Human African trypanosomiasis of the CNS: current issues and challenges. J Clin Invest 113: 496-504.

41. Ngwa GA, Shu WS (2000) A mathematical model for endemic malaria with variable human and mosquito populations. Math Comput Model 32: 747-763.

42. Gürtler RE, Cecere MC, Castanera MB, Canale D, Lauricella MA, et al. (1996) Probability of infection with *Trypanosoma cruzi* of the vector *Triatoma infestans* fed on infected humans and dogs in northwest Argentina. Am J Trop Med Hyg 55: 24-31.

43. Michalsky EM, Rocha MF, da Rocha Lima AC, França-Silva JC, Pires MQ, et al. (2007) Infectivity of seropositive dogs, showing different clinical forms of leishmaniasis, to *Lutzomyia longipalpis* phlebotomines sand flies. Vet Parasitol 147: 67-76.

44. van den Hurk AF, Nisbet DJ, Hall RA, Kay BH, MacKenzie JS, et al. (2003) Vector competence of Australian mosquitoes (Diptera: Culicidae) for Japanese encephalitis virus. J Med Entomol 40: 82-90.

45. Machado EM, Fernandes AJ, Murta SM, Vitor RW, Camilo DJ Jr, et al. (2001) A study of experimental reinfection by *Trypanosoma cruzi* in dogs. Am J Trop Med Hyg 65: 958-965.

46. World Health Organisation. Available: http:/www.who.int/mediacentre/factsheets/fs117/en. Accessed 3 November 2011.

47. Gould EA (2001) Flavivirus infections in humans. Encyclopedia of Life Sciences.

48. Bora D (1999) Epidemiology of visceral leishmaniasis in India. Natl Med J India 12: 62-68.

49. Ministério de Saúde (1999) Enfermedades Infecciosas y Parasitarias: guίa de bolso. Brasίlia, Brazil. 332 p.

50. Brun R, Blum J, Chappuis F, Burri C (2010) Human African trypanosomiasis. The Lancet 375: 148-159.

51. Teixeira AR, Nitz N, Guimaro MC, Gomes C, Santos-Buch CA (2006) Chagas disease. Postgrad Med J 82: 788-798.

52. Pépin J, Milord F, Khonde AN, Niyonsenga T, Loko L, et al. (1995) Risk factors for encephalopathy and mortality during melarsoprol treatment of *Trypanosoma brucei gambiense* sleeping sickness. Trans R Soc Trop Med Hyg 89: 92-97.

53. The Center for Food Security and Public Health (2007). JAPE_A2007. Ames, Iowa: The Center for Food Security and Public Health. Available: http://www.cfsph.iastate.edu/Factsheets/pdfs/japanese_encephalitis.pdf. Accessed 3 November 2011.

54. Centre de Coopération Internationale en Recherche Agronomique pour le Développement (2011). Japanese Encephalitis. Available: http://pigtrop.cirad.fr/content/pdf.984. Accessed 3 November 2011.

55. Courtenay O, Quinnell RJ, Garcez LM, Shaw JJ, Dye C (2002) Infectiousness in a cohort of Brazilian dogs: why culling fails to control visceral leishmaniasis in areas of high transmission. J Infect Dis 186: 1314-1320.

56. Nunes CM, Lima VM, Paula HB, Perri SH, Andrade AM, et al. (2008) Dog culling and replacement in an area endemic for visceral leishmaniasis in Brazil. Vet Parasitol 153: 19-23.

57. Eloy LJ, Lucheis SB (2009) Canine trypanosomiasis: etiology of infection and implications for public health. J Venom Anim Toxins incl Trop Dis 15: 589-611.

58. Nuraini N, Soewono E, Sidarto KA (2007) Mathematical model of dengue disease transmission with severe DHF Compartment. Bull Malays Math Sci 30: 143-157.

59. Otero M, Solari HG (2010) Stochastic eco-epidemiological model of dengue disease transmission by *Aedes aegypti* mosquito. Math Biosci 223: 32-46.

60. Watt G, Jongsakul K (2003) Acute undifferentiated fever caused by infection with Japanese encephalitis virus. Am J Trop Med Hyg 68: 704-706.

61. Alvar J, Bashave S, Argaw D, Cruz I, Aparicio P, et al. (2007) Kala-azar outbreak in Libo Kemkem, Ethiopa: epidemiologic and parasitologic assessment. Am J Trop Med Hyg 77: 275-282.

62. Checchi F, Filipe JA, Haydon DT, Chandramohan D, Chappuis F (2008) estimates of the duration of the early and late stage of gambiense sleeping sickness. BMC Infect Dis 8:16.

63. Rassi A, Marcondes de Rezende J, Luquetti AO, Rassi A Jr (2010) Clinical phases and forms of Chagas disease. In: American trypanosomiasis: Chagas disease one hundred years of research. Tibayrenc M, Tellería J, editors. Elsevier. pp. 711-715.

64. Ueba N, Kimura T, Nakajima S, Kurimura T, Kitaura T (1978) Field experiments on live attenuated Japanese encephalitis virus vaccine for swine. Biken J 21: 95-103.

65. Scherer WF, Moyer JT, Izumi T (1959) Immunologic studies of Japanese encephalitis virus in Japan. V. Maternal antibodies, antibody responses and viremia following infection of swine. J Immunol 83: 620-626.

66. Palatnik-de-Sousa CB, Batista-de-Melo LM, Borja-Cabrera GP, Palatnik M, Lavor CC (2004) Improving methods for epidemiological control of canine visceral leishmaniasis based on a mathematical model. Impact on the incidence of the canine and human disease. Anais da Academia Brasileira de Ciências 76: 583-593.
